# Microclimatic changes caused by plant invasions and warming: uncovering thermal costs and benefits to a tortoise

**DOI:** 10.1093/conphys/coaf016

**Published:** 2025-03-05

**Authors:** Raquel A Garcia, Susana Clusella-Trullas

**Affiliations:** Department of Botany and Zoology, Centre for Invasion Biology, Stellenbosch University, Merriman Street, Stellenbosch 7600, Western Cape Province, South Africa; Department of Botany and Zoology, Centre for Invasion Biology, Stellenbosch University, Merriman Street, Stellenbosch 7600, Western Cape Province, South Africa; School for Climate Studies, Stellenbosch University, Merriman Street, Stellenbosch 7600, Western Cape Province, South Africa

**Keywords:** Behavioural thermoregulation, buffering and amplifying capacity, chelonian, climate change, exotic plants, mechanism, operative temperature, spatial thermal heterogeneity, thermal habitat quality, thermal performance curve

## Abstract

Non-native plant invasions and climate warming alter the microclimatic conditions that organisms experience in their habitats, with potential implications for the fitness of native faunal species, particularly ectotherms. Predictions for species conservation increasingly use microclimate data at fine spatial scales relevant to organisms, but they typically overlook the modulating effect that vegetation changes have on the microclimates available in the habitat. Here we quantify the microclimatic changes imposed by invasive trees and simultaneous warming on native habitats and assess the resulting thermal benefits and costs to a small tortoise species (*Homopus areolatus*) from an organismal perspective and throughout its life cycle. We logged operative temperature above- and belowground in the field, covering the diversity of microhabitats across the four seasons of the year, and assessed the species’ optimal temperature in the laboratory. Moving beyond the common use of averages, we applied a range of metrics to quantify differences between invaded and native areas in spatio-temporal temperature distributions, combined effects with warming and thermal habitat suitability for the species. We found that invaded areas became cooler and less exposed to temperatures above the species’ optimal in summer. This buffering effect is expected to become more pronounced with further climate warming, turning invaded areas into potential thermal refugia. However, reduced spatial thermal heterogeneity during warm periods, more prevalent sub-optimal low temperatures in winter and colder underground incubation conditions in invaded areas could be detrimental to the species’ long-term performance. Our results reveal the mixed nature of thermal effects of invasive plants on ectotherms, underscoring the importance of applying a suite of metrics to assess microclimate distribution changes. The approach used here illustrates the value of integrating thermal physiological and microclimatic information for a more mechanistic understanding of conservation problems.

## Introduction

Making accurate predictions for species conservation requires explicit incorporation of microhabitat data at spatio-temporal scales relevant to the species’ body size and ecological preferences (Levin, 1991; Potter *et al.*, 2013; Scheffers *et al.*, 2014; Kemppinen *et al.*, 2024). Ectothermic animals are especially reliant on fine-grained thermal variation in their habitats, as they shuttle among available microsites with different temperatures for behavioural thermoregulation (Huey, 1991). By thermoregulating effectively, animals can maintain optimal body temperatures and thus maximize activity time, with positive impacts on fitness and survival (Porter *et al.*, 1973; Gunderson and Leal, 2016). Use of fine-scale data capturing the heterogeneity of microclimates has increased over the last decades in species distribution and biophysical modelling (Lembrechts *et al.*, 2019; Briscoe *et al.*, 2023). Many such models account for microclimatic changes over time driven by global warming trends (e.g. Mitchell *et al.*, 2008). By contrast, temporal changes in key habitat features that also modulate microclimatic conditions, such as vegetation or topography, are typically overlooked (Gunderson, 2023; Stark *et al.*, 2023). Vegetation plays a crucial role in shaping spatial thermal heterogeneity (Sears *et al.*, 2011; Pincebourde and Suppo, 2016), with higher diversity in plant structure creating larger variation in temperature within landscapes or vegetation patches (Londe *et al.*, 2020). At the same time, vegetation also buffers extreme temperatures (De Frenne *et al.*, 2019), accounting for differences in daily maximum temperatures of up to 5°C between the forest understory and open areas (Chen *et al.*, 1999).

From habitat conversion and degradation to afforestation and the introduction of invasive non-native plants, many anthropogenic threats to biodiversity imply changes in vegetation and, consequently, in the thermal landscapes available to resident fauna (Frishkoff *et al.*, 2015; Tuff *et al.*, 2016; Nowakowski *et al.*, 2018; Garcia and Clusella-Trullas, 2019). Altered composition and physical structure of plant communities in invaded landscapes lead to thermal changes, affecting populations of ectothermic species (Garcia and Clusella-Trullas, 2019). The numbers of non-native plants recorded across the globe are rising (Seebens *et al.*, 2017; Pyšek *et al.*, 2020,), and anthropogenic warming has continued unabated over the last decades (IPCC, 2023). Whereas there has been research into how warming might assist further plant invasions (e.g. Liu *et al.*, 2017), less attention has been focused on how the two threats act simultaneously to shape thermal landscapes for ectothermic animals (but see, e.g. Huang *et al.*, 2014; Stark *et al.*, 2023).

South Africa’s biodiversity is threatened with both non-native plant invasions and climate change (Ziervogel *et al.*, 2014; van Wilgen *et al.*, 2022). Nearly 800 species of non-native plants, more than half of which are trees and shrubs, have become established across the country (Richardson *et al.*, 2020) with consequences for native ectothermic species’ abundance and diversity (Clusella-Trullas and Garcia, 2017). At the same time, mean and maximum temperatures have increased over the last five decades in South Africa, and this trend is expected to continue up to 2050 and beyond (MacKellar *et al.*, 2014). Several groups of ectotherms are at potential risk from these two threats (Loehr, 2012; Botts *et al.*, 2013; Schreuder and Clusella-Trullas, 2017), including tortoises (family *Testudinidae*), a diverse group in South Africa and highly endemic in the Cape region of the country (Branch *et al.*, 1995). Populations of tortoises and turtles have been declining worldwide (Stanford *et al.*, 2020). Some of the life-history characteristics of tortoises, such as herbivory and long lifespan, and their habitat structure needs for shelter from predators and heat, increase their vulnerability to non-native plant invasions (Stewart *et al.*, 1993; Gray and Steidl, 2015). In turn, their low dispersal capacity, occurrence in fragmented habitats, and temperature-dependent sex determination make them susceptible to climate change (Fernández-Chacón *et al.*, 2011; Ihlow *et al.*, 2012).

The parrot-beaked dwarf tortoise or common padloper (*Homopus areolatus*, Thunberg, 1787) is endemic to South Africa. It is restricted to habitats that are often invaded by non-native plants including tree species in the genera Acacia, Pinus and Eucalyptus, which are among the most impactful invaders in South Africa (Richardson *et al.*, 1989, 2003; Wells *et al.*, 2023). With growth forms contrasting with the native shrubland vegetation, these invasive trees severely alter the habitat structure (Martin and Murray, 2011) and associated temperature distribution. Although the small body size of *H. areolatus* (males <100 mm; Branch, 1998) is an advantage for accessing microclimatic refugia (Pincebourde *et al.*, 2021; Comte *et al.*, 2024), the associated low thermal inertia and high risk of overheating increase their vulnerability to warming (Perrin and Campbell, 1981; Hailey and Coulson, 1996).

Here, we explore the effects of invasive non-native trees on the thermal landscapes available to *H. areolatus* in four sites within the species’ range in South Africa. Using operative environmental temperature data collected in the field, we compare the thermal landscapes available to organisms in native habitats with and without invasive Acacia, Pinus or Eucalyptus trees. First, we describe the temporal composition and spatial heterogeneity of the thermal landscapes. Second, we assess their suitability to *H. areolatus*, based on this study’s empirical data on the species’ thermal physiology. Third, we analyse the buffering or amplifying capacity of microsites relative to air temperatures as an indication of the combined effect of plant invasions and global warming trends. We hypothesize that the thermal landscapes in invaded areas will be cooler, more homogeneous and less suitable for the species, and that the cooling effect would become beneficial in warming scenarios. Our results highlight the thermal challenges imposed by non-native vegetation and simultaneous warming trends on ectothermic organisms and illustrate the value of integrating thermal physiology and microclimates for guiding conservation.

## Materials and Methods

Our study involved a combination of fieldwork, laboratory experiments and statistical analyses. The field and laboratory experiments followed standard protocols to ensure animal welfare and reduce stress. Our work was conducted under the Cape Nature Permit AAA041–00166–0056 and animal ethics clearance from the Stellenbosch University’s Research Ethics Committee: Animal Care and Use (SU-ACUD16–00188). The statistical analyses were conducted using R (version 4.3.1; R Core Team, 2023). We report the mean or median ± standard deviation unless stated otherwise, and we set the significance level at α < 0.05.

### Study system

*Homopus areolatus* has a wide distribution covering mostly the winter rainfall regions of the Fynbos biome in the coastal lowland of the Western and Eastern Cape provinces, with relic populations inland at higher altitudes (Bates et al., 2014; Branch, 1998). The species is highly associated mainly with renosterveld, a vegetation unit of the Fynbos Biome that is dominated by small evergreen shrubs, especially renosterbos *Elytropappus rhinocerotis* (Mucina *et al.*, 2005). In renosterveld habitat, *H. areolatus* can find low dense vegetation to escape predators and extreme temperatures (Hofmeyr and Keswick, 2018). Classified as ‘Least Concern’ on the IUCN Red List, and widely distributed and abundant in some areas, *H. areolatus* is a viable species for field monitoring. However, its population is decreasing (Hofmeyr and Keswick, 2018) as large areas of their habitat are threatened with agricultural intensification and plant invasions (Topp and Loos, 2019).

Females typically reach a carapace length of 120 mm and a weight of up to 300 g, whereas males measure only up to 100 mm and weigh up to 140 g (Branch, 1998). *Homopus areolatus* females lay one or more clutches of two to three eggs in nest holes dug in the soil, between mid-winter and early summer, and hatching occurs 150–320 days later with the first rains in early autumn (Branch, 1989; personal communication from Dr Hofmeyr, University of the Western Cape). Females reach maturity at the age of 8–10 years and individuals can live >28 years in captivity (Branch, 1989). *Homopus areolatus* remains active throughout most of the year in our study region (Boycott and Bourquin, 2000). They are thought to have a generalist diet: in the wild they forage most herbaceous species that grow low on the ground (Hofmeyr and Keswick, 2018; personal communication from Dr Hofmeyr, University of the Western Cape) and in captivity they accept a wide range of food (Branch, 1989).

**Figure 1:**
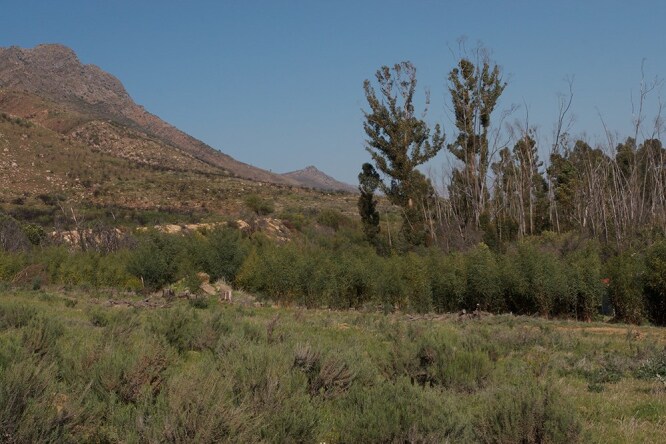
**Native and invaded areas*.*** Example of a study site with native renosterveld habitat in the foreground and invasive non-native Acacia and Eucalyptus trees in the background. The age and spatial coverage of the invasive trees varied among study sites.

Our field sites were four private farms in the winter-rainfall area of South Africa’s Western Cape Province, an area that is particularly affected by plant invasions (Van Wilgen *et al.*, 2023). The four sites comprise *H. areolatus* tortoises and were distributed as pairs along the latitudinal range of our study species. The conservancy of the De Tuin private farm (33° 07′ 58.4“ S, 18° 58’ 47.8” E, ‘De Tuin’) and the Waterval Private Nature Reserve (32° 59′ 07.0“ S, 19° 01’ 32.2” E, ‘Watervale’), both in Porterville, were the northern edge pair; a private farm 10 km north of Malmesbury (33° 22′ 12” S, 18° 42′ 19″ E, ‘Malmesbury’) and the Telkom Conservancy area in Klipheuwel (33° 41′ 43” S, 18° 42′ 14″ E, ‘Klipheuwel’) were the southern edge pair. All sites had native renosterveld but they all had distinct stands of non-native *Acacia saligna* and some of them also had *Eucalyptus globulus*, *Pinus sp*. or *Acacia mearnsii* (Fig. 1). Within each site we considered two areas: ‘native areas’ as those dominated by native vegetation and ‘invaded areas’ as those with stands of invasive trees. We conducted our fieldwork between July 2017 and August 2018 and considered the months of June–August as winter, September–November as spring, December–February as summer, and March–May as autumn.

### Righting response measurement

We collected individuals of *H. areolatus* in two sites covering the species’ latitudinal range, De Tuin and Malmesbury, in two seasons: 40 individuals in spring (September–November 2017) and 26 in autumn (April–June 2018) of which four were recaptures from spring. All individuals were marked with scute notches following Ernst (1974) and their weight and carapace dimensions were measured in the field. We recorded the capture locations for release after the trials. Each individual was placed in a plastic container lined with cloth and perforated in multiple places to allow ample ventilation, and all containers were transported to the laboratory inside insulated coolers. In the laboratory, the individuals were kept in large plastic boxes (dimensions ~75 (Length) × 40 (Width) × 40 (Height) cm) with sand and rocks to mimic the natural habitat and fitted with a heat lamp and a UVB lamp programmed to follow the natural light cycle. They were fed vegetables and given water *ad libitum*.

To describe the thermal dependence of relevant species’ traits, we assessed the effect of temperature on the individuals’ ability to right themselves, i.e. on the time it took for an individual to return to a prone position after being overturned. In their natural habitat, when walking over uneven terrain and bushes or during male fights, *H. areolatus* individuals often fall over on their carapaces. Upside down, individuals become vulnerable to predation, overheating, desiccation or starvation (Steyermark and Spotila, 2001; Delmas *et al.*, 2007), but they are naturally motivated to right themselves using their limbs and neck (Burger, 1976; Freedberg *et al.*, 2004). The speed of righting response thus has direct impacts on fitness and population growth, making it an ecologically relevant measure of performance. Indeed, righting speed is commonly used as an indicator of fitness on armoured animals including chelonians (Steyermark and Spotila, 2001; Delmas *et al.*, 2007; Golubović *et al.*, 2013).

We conducted the righting response trials following a period of captivity of up to 40 days depending on the specific capture date and the length of the thermal preference trials that preceded our trials. Before the righting response trials, the individuals were fasted for 6 days to avoid variation in absorptive state during the trials. A total of 66 individuals (47 adults, 9 young adults or juveniles and 12 of uncertain age) underwent the trials: 34 from De Tuin and 32 from Malmesbury (Table S2). Following published methods for organisms including turtles and tortoises (Steyermark and Spotila, 2001; Delmas *et al.*, 2007; Bozinovic F. *et al.*, 2016), we conducted righting response trials in a temperature-controlled room at six temperatures from 10 to 35°C at 5°C intervals. We used terraria with sufficient space for the animals to move (dimensions ~25 (Length) × 22 (Width) × 20 (Height) cm) and covered the bottom of the terraria with coarse sand paper to provide sufficient grip. Immediately before starting the trial, we measured the body temperature of the individuals by placing a thin thermocouple inside the neck fold where the head retracts inside the shell cavity. This method was less intrusive than measuring cloacal temperature and, for small tortoises, it has been shown to provide similar measurements to cloacal or core body temperatures (Geffen and Mendelssohn, 1989). We then placed one individual inside each terrarium and left the individuals to equilibrate to the target temperature for 1 h.

The trial’s experimental period started when individuals were turned upside down. We video-recorded the individuals for the duration of the trial and monitored them every 15 min. If after two consecutive periods of 15 min an individual had not righted itself, its original position was restored for 15 min, after which we started the trial again. The trial ended when an animal had successfully righted itself three times, or when it had failed to right itself after two consecutive periods of 30 min separated by a 15-min rest break. We ran the trials separately for each site, with two groups of individuals in the spring season and one group in the autumn season. We followed a sequence of temperatures from those expected to be most optimal, 20 and 25°C, to 15 and 30°C and, finally, the most extreme treatments, 10 and 35°C, except for one group of individuals (10 out of 66) for which 10°C preceded 20°C. We performed two trials per day except when a particular treatment was lengthier or shorter than expected (for 27 out of 66 individuals). We undertook the trials during the active period of the photophase (between 06:00 h and 18:00 h) and allowed the individuals to rest between trials for at least 1 h and up to 5 h.

We scanned the video footage to estimate the individuals’ righting speed using the motion analysis software Kinovea. Following a latency period, when individuals were immobile, they attempted to right themselves by actively moving the neck and limbs. The latency time varied across individuals and partly represented their motivation to right themselves, whereas the righting time represented the ability of individuals to right themselves based mainly on their physical traits and physiological state (Steyermark and Spotila, 2001; Elnitsky and Claussen, 2006; Polo-Cavia *et al.*, 2012). Righting time was thus the trait of interest for assessing performance. As righting bouts were often interrupted by further periods of immobility, we considered only the last righting attempt where the individual successfully righted itself. Thus, if the individual interrupted its righting attempt for >5 s, we reset our clock to zero and started timing the following righting attempt. We measured the ‘successful righting time’ as the time elapsed between the start and the end of the successful righting attempt. For each individual, we measured this trait to the nearest 0.01 s and retained all measurements across repetitions and treatment temperatures but removed zero values that corresponded to absent or failed righting attempts. To quantify the performance or rate of righting, we computed the inverse of the successful righting time (‘righting speed’). We used the mean righting speed over all repetitions for each combination of individual, temperature treatment, season and site. The resulting righting speed data provide information about the thermal tolerance of adults and juveniles. We were, however, unable to find published estimates of thermal tolerance for the embryonic stage.

### Thermal performance curves

After inspecting the righting speed data for potential outliers, we performed linear mixed-effects modelling (LMM) using the ‘lme4’ (Bates *et al.*, 2015) and ‘lmerTest’ (Kuznetsova *et al.*, 2017) packages to assess potential effects of site, season or sex on performance traits. Model selection (Zuur *et al.*, 2009) started with a full, complex model with all possible and meaningful fixed effects and interactions as well as meaningful random slopes and intercepts. Our dependent variable was mean righting speed, the fixed effects included body temperature (polynomial), site, season and sex and the random effect was tortoise ID. We then compared the full model with models containing the same fixed effect structure but alternative random effect structures, using restricted maximum likelihood (REML) estimation. Once the best random model was identified, we compared different fixed effect structures, maintaining the selected random effects and using maximum likelihood (ML) estimation, to arrive at the final model. We relied on Akaike Information Criteria (AIC; ‘AICcmodavg’ package; Mazerolle, 2023) to compare models and verified model assumptions by plotting residuals versus fitted values. The final model was refitted using REML estimation.

As our final LMM indicated that there was no effect of site, season or sex on righting speed (Table S3), we pooled all the data together and opted for fitting a single thermal performance curve for *H. areolatus*, with mean righting speed data versus body temperature, using the *rTPC* package in R (version 1.02; Padfield and O’Sullivan, 2021). After removal of one outlier point, we had 233 non-zero data points throughout the six temperature treatments (Table S4).

Of the 24 models available in the *rTPC* package, our list of candidate models was based on a set of three criteria (Adams *et al.*, 2017; Low-Décarie *et al.*, 2017; Padfield *et al.*, 2021). First, the model provided the expected theoretical shape, including asymmetry with respect to the optimum temperature. Second, to reduce the chances of overfitting, the model had no more than four free parameters, thus not exceeding the number of points available for fitting nor the number of temperatures measured. Third, to increase biological meaning, the model had parameters that were stable, physically interpretable and transferable and, whenever possible, included our parameters of interest (optimal temperature and the upper temperature limit of the righting response). We fitted the 12 models that met these criteria (Table S5) and selected the one with the lowest corrected AIC (AICc). From the curve we extracted the optimal temperature and the temperature range at which performance was maintained at 80% of peak performance (thermal suitability breadth B80), but we were not able to extract the upper temperature limit at which righting response was null (see Results).

### Operative temperature sampling

To characterize the thermal landscapes available in the study sites, we collected operative temperature (OTM) data in both the native and invaded areas of the four sites using simplified physical models of OTMs of *H. areolatus*. The OTMs were soft drink cans (150 and 330 ml) painted with a colour that matched the tortoise carapace’s reflectance (see Supplementary Methods) and fitted with iButton temperature loggers (DS1921G-F5#/MAXIM Thermochron, −40 to +85°C, accuracy of ±1°C) suspended in the centre of the cans. The loggers were secured at the end of a wooden stick held in place inside the can at the other end by a cork that sealed the can opening. To ensure that the OTMs remained in place, they were tied with rope to nearby bushes or to sticks inserted in the ground. The OTMs were validated against live animals, tortoise carcasses and copper replicas of carcasses (see Supplementary Methods).

To sample the diversity of microclimates, we placed the OTMs in the field using a randomized block design. In both native and invaded areas in each site, we first defined the main broad native habitat units present and distributed five quadrats (~1 × 1 m) among these units to ensure proportional coverage. In each quadrat we placed three to four OTMs at ground level, ensuring representativeness of microsites that differed in exposure to the sun. The different microsites were: fully exposed to the sun; partly exposed to the sun, at the edge of vegetation; in the shade or at least protected from the northern sun; and inside thick vegetation. The availability of each microsite type differed between habitats, with native areas being dominated by fully or partly exposed microsites and invaded areas by shaded and partly exposed microsites. We also logged operative soil temperatures underground (hereafter referred to as ‘underground temperature’) to assess available conditions for incubation, which occurs at a depth of 6–8 cm in holes excavated by female tortoises (Boycott and Bourquin, 2000). In both native and invaded areas in all sites, we buried one iButton, enclosed in a metal case, in three of the quadrats at ~8 cm belowground under vegetation. In one site (Malmesbury) we added five OTMs inside burrows. We selected burrows that were used by *H. areolatus* individuals or that appeared suitable for the species and placed the OTM inside until it was completely shaded but still visible from the outside. In each site, we thus had a minimum of 20 temperature loggers (17 on the ground and three buried in the soil), with an extra five loggers placed in burrows in one site. In the four study sites we also measured air temperature in the native habitat by deploying an iButton suspended ~1.2 m above the ground and protected from direct sun radiation by a ventilated plastic container.

The iButtons logged hourly temperature for just over a year (between July 2017 and August 2018). They were programmed before deployment and reprogrammed in the field every 3 months before reaching their storage capacity. We cleaned the raw data by removing 1 h before and after logger deployment or reprogramming in the field, as the handling of OTMs might have affected the data. We then conducted a visual inspection of the data to remove outliers due to possible logger malfunctioning or disturbance and removed 0.1% of the raw data (1640 data points).

Our final dataset had daytime hourly operative temperature between 07:00 h and 19:00 h for a whole year from 1 August 2017 to 31 July 2018. In total we had data for 167 OTMs across the four study sites logging for an average of 4302 h (Table S6). The small variation in the total logging period across OTMs was due to logger malfunctioning, loss of iButtons and, in the Malmesbury site, a fire that affected two quadrats in the summer of 2018. The cleaned data were categorized according to site, season, habitat type (native or invaded), OTM, quadrat and type of temperature (ground, underground, burrow or air).

### Thermal landscape changes

We used the operative temperature data to characterize the thermal landscapes of both native and invaded areas across sites and seasons. Our metrics were computed for each site, habitat and quadrat and, depending on the metric, for each season, day or hour of the day, but we then averaged the results to focus our discussion on differences between habitat types and among seasons. First, to describe the thermal composition, we calculated the daily median, minimum, maximum and range of temperature for either ground or underground microsites. To assess the spatial heterogeneity of ground thermal landscapes we computed, for each hour of recorded data, the range of temperature values among OTMs in each quadrat (Pincebourde *et al.*, 2016; Aalto *et al.*, 2022).

Second, we assessed the suitability of ground microsites for *H. areolatus* based on the species’ thermal suitability breadth B80 estimated above. Assuming operative temperatures were optimal when they fell within B80, we computed the daily time when at least one microsite within a quadrat was optimal. To assess exposure to potentially detrimental temperatures, we computed the daily time when at least half of the microsites within a quadrat were above the upper end of the B80 range or below the lower end of the B80 range. To assess the spatial ratio of optimal and non-optimal microsites, we computed the percentage of OTMs across a quadrat that were within, below or above the B80 range. We also calculated the thermal habitat quality index *de* following the approach of Hertz et al. (1993) but using the species’ thermal suitability breadth B80 instead of thermal preference as in the original formulation. Our *de* index was the mean of the absolute values of the deviation of microsite temperatures from B80, using the lower bound of B80 when the temperature was below B80 and the upper bound otherwise.

Third, as a measure of the potential combined effect of non-native plant invasions and warming, we assessed the potential for microsites in invaded and native areas to decrease (buffer) or increase (amplify) air temperatures (Ewers and Banks-Leite, 2013; Varner and Dearing, 2014; Locosselli *et al.*, 2016; Rita *et al.*, 2021; Gril *et al.*, 2023). This assessment compared native and invaded habitats and was conducted for ground and underground microsites separately. To calculate our metrics, we built linear mixed-effects models for each site, habitat, quadrat and season combination, following the same protocol described for the righting speed data. The LMMs had hourly microsite temperature as the response variable and hourly air temperature as the explanatory variable, with day as a random effect (Rita *et al.*, 2021; Gril *et al.*, 2023). LMM slopes different from one indicated that microsite temperatures behaved more independently from air temperatures, either buffering or amplifying them (slope smaller or larger than one, respectively). To quantify the total modifying capacity for each site, habitat, quadrat and season combination, we summed the individual areas when the fitted line was above and below the unity line (amplifying or buffering capacity, respectively, with the buffering capacity assuming negative values). Negative total modifying capacity values thus indicated that the buffering effect was larger than the amplifying effect, and positive values the opposite case (Davis *et al.*, 2019).

Finally, we assessed differences among habitat types and seasons for each of the above thermal landscape metrics (median, maximum and minimum temperature; temporal and spatial range of temperature; time within, above or below B80; thermal habitat quality *de*; and total buffering and amplifying capacity) using LMMs and following the same protocol as above. For each of the metrics (continuous response variables) calculated for each site, quadrat and day, we analysed the effect of habitat type and season (fixed categorical predictors), with quadrat and site identity as random factors.

## Results

### Thermal performance curves

Body temperature measured before the start of the righting trials closely matched treatment temperatures, with the exception of the coldest treatment, indicating adequate equilibration to the treatment temperatures (medians and standard deviations of body temperature for the 10, 15, 20, 25, 30 and 35°C treatments: 14.6 (1.8)^o^C, 16.8 (1.9)^o^C, 20.9 (0.7)^o^C, 24.6 (1.0)^o^C, 27.5 (1.1)^o^C and 32.4 (1.1)^o^C, respectively; Fig. S4).

**Figure 2:**
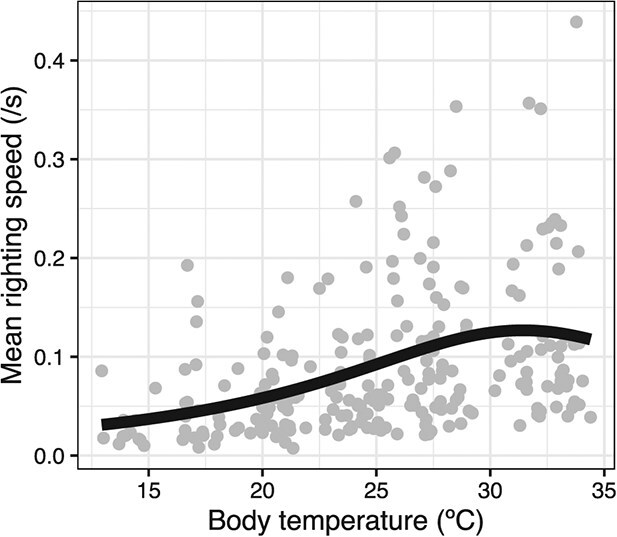
**Righting response curve for *H. areolatus.*** The solid line is the fitted line for the thermal response curve of the righting performance for *H. areolatus* showing mean righting speed as a function of body temperature (righting speed = 1/(1 + 76.79 + −4.45 × temp + 0.07 × temp^2^)). The grey circles represent the data points (means across repetitions) for all individuals collected from the four sites in spring and autumn.

Of the biologically realistic curves we fitted for *H. areolatus* (all sites, seasons and sexes pooled together), Flinn’s model (Flinn, 1991) had the lowest AICc and was thus selected (Table S5). The curve showed the righting speed increasing with body temperature up to a peak performance of 0.13 s^−1^ at a temperature of 31.4°C (optimal temperature), after which it declined (Fig. 2). The temperature boundaries of the thermal suitability breadth B80 were 26.2 and 34.4°C. Although there was a decreasing trend at temperatures above the optimal temperature, our range of temperature treatments was deemed insufficient to provide reliable estimates of the upper temperature limit of righting response.

### Changes in the composition of thermal landscapes

**Figure 3:**
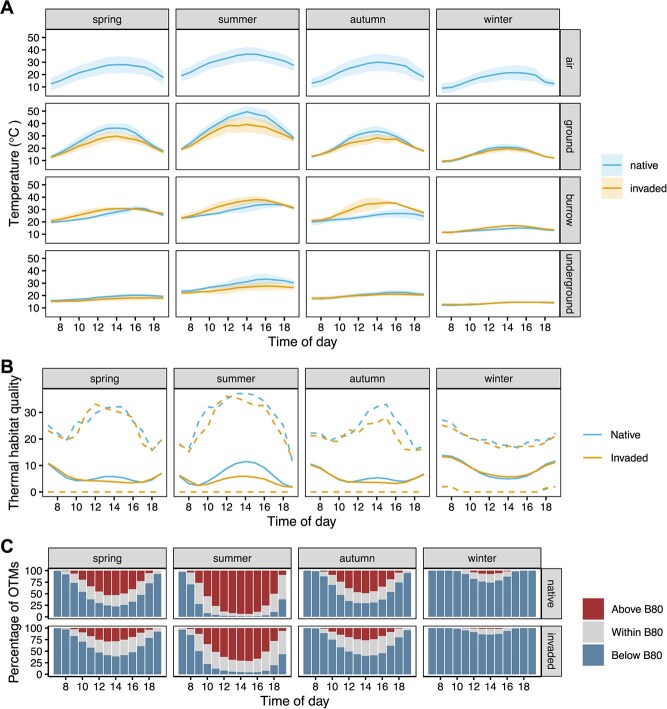
**Operative temperature and thermal habitat suitability for *H. areolatus* across the day in native and invaded areas. A)** For each season, the plots show the median (solid blue and orange lines for native and invaded areas, respectively) and standard deviation (polygons around the lines) of hourly temperature in ground and underground microsites, as well as in burrows when available. Air temperature in native areas is also shown. operative temperature corresponds to hourly medians and standard deviation across all sites, quadrats and days for ground and underground microsites, across all sites, OTMs and days for burrows, and across all sites and days in the native areas for air temperature. See Fig. S5 for plots per site. **B)** The thermal quality index *de* (Hertz *et al.* 1993) for *H. areolatus* is based on the B80 thermal suitability breadth derived from the thermal performance curve for the righting response of *H. areolatus*. For all sites pooled together and each season, the mean (solid lines) and minimum and maximum (dashed lines) *de* values are shown across time for both native (blue) and invaded (orange) habitats. *de* values closer to zero correspond to better quality thermal habitats. See Fig. S7 for plots per site. **C)** Percentage of OTMs within, above and below *H. areolatus*’ B80 thermal suitability breadth, based on the thermal performance curve for righting response for the species, and averaged across sites, quadrats and days. See Fig. S8 for plots per site.

For most thermal landscape metrics, habitat type explained more variation in summer than in the rest of the year. Ground operative temperatures were generally lower in invaded areas compared to native areas (Fig. 3A). This difference held across the four microsite types varying in sun exposure (Fig. S6). Among the four study sites, summer daily medians averaged across days and quadrats ranged from 30.8 (3.2)^o^C to 34.8 (4.7)^o^C in invaded areas (Table S7) and were lower than native areas (*P* < 0.001, Table S8), which ranged from 37.2 (3.3) to 39.6 (2.4)^o^C. Native areas among the four sites experienced wider daily ranges of temperature than invaded areas (Table S7). This difference in daily ranges between the two habitats was largest in summer (7.4°C, varying from 6.8 to 8.0°C, *P* < 0.001, Table S9), when daily ranges in native areas reached 39.8 (3.2)^o^C. Compared to native areas, daily minimum temperatures in invaded areas were slightly lower in summer but higher in winter and autumn (Supplementary Table S10). In turn, invaded areas experienced lower daily maximum temperatures than native areas throughout the year (difference between the two habitats in summer of 7.7 (7.1–8.3)^o^C, *P* < 0.001, Supplementary Table S11). In fully exposed microsites, summer maximum temperature averaged 54.5°C (standard variation 2.6°C) and 48.7°C (3.9°C) in native and invaded areas, respectively (Fig. S6).

Underground temperatures were buffered in both habitats, with daily medians ranging among sites and habitat types from 13.0 (0.3)^o^C to 30.0 (1.3)^o^C (Fig. 3A and Supplementary Table S12). However, underground microsites were cooler in habitats with invasive trees than without them for most of the year (largest difference in daily medians between the two habitats, recorded in summer, of 3.2°C (2.9–3.5°C), *P* < 0.001, Supplementary Table S13). Underground microsites were also more stable throughout the day in invaded areas, with the largest difference in daily temperature range, relative to native areas, occurring in summer (difference of 3.1 (2.7 to 3.4)^o^C, *P* < 0.001, Supplementary Table S14). In both habitats, however, there was a steeper daily increase in underground temperature in summer than in other seasons, reaching maximum values of 35.5 (2.9)^o^C and 32.5 (9.2)^o^C in native and invaded areas, respectively (Supplementary Table S12). Daily median temperatures in burrows were lower than ground microsites and reached a peak later in the day (Fig. 3A; Supplementary Table S15). However, daily median burrow temperatures were higher in invaded areas than in native areas (*P* < 0.001, Supplementary Table S16), with summer medians of 33.8 (3.2)^o^C and 30.5 (1.5)^o^C, respectively.

### Changes in the spatial heterogeneity of thermal landscapes

**Figure 4:**
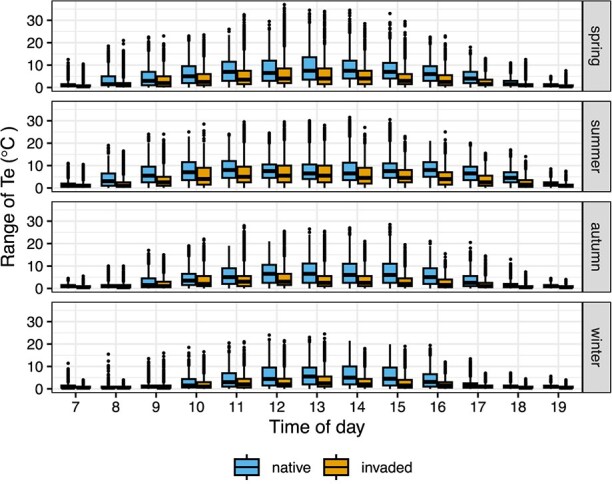
**Spatial range of operative temperatures across the day.** For each hour of daytime, the boxplots show the distribution of values of the spatial range of temperatures, calculated per quadrat, for all quadrats, sites and days. The data are shown for native (blue) and invaded (orange) habitats in each season (rows).

The two habitats differed in the range of microsite types and associated microclimates available across space at any given time, with native areas offering a wider spatial range of operative temperatures during most of the day (Fig. 4; Table S7). At the peak of the heat (most frequently ~14:00 h throughout the year), when spatial thermal heterogeneity is crucial for behavioural thermoregulation, the presence of invasive trees decreased the spatial thermal range by 2.5°C (2.2–2.8°C, *P* < 0.001, Supplementary Table S17).

### Changes in thermal landscape suitability to *H. areolatus*

The thermal habitat quality index *de* varied throughout the day, with thermal quality increasing (*de* becoming smaller) during the morning and decreasing in the afternoon in all seasons, and the middle of the day showing the highest quality in winter but a dip in the remaining seasons (Fig. 3B). Compared to native areas, invaded areas had higher thermal quality in the daily peak of the heat in all seasons except for winter (Supplementary Table S18). The average daily time when there was at least one optimal microsite (within B80) in a quadrat was higher in native areas than in invaded areas in autumn through to spring, ranging among sites from 108 to 300 min of daily optimal time (averages of daily time across days and quadrats, Supplementary Table S19). In summer, however, *H. areolatus* individuals in invaded areas had 84 min more daily optimal time (66–102 min, *P* > 0.001, Table S20) than in native areas and were exposed to 232 min less of sub-optimal hot temperatures (102–162 min, *P* < 0.001, Table S21). By contrast, exposure to sub-optimal cold temperatures was longer in invaded areas throughout the year, with the largest difference between the two habitats occurring in spring (*P* < 0.001, Table S22).

### Changes in the amplifying and buffering capacity of thermal landscapes

**Figure 5:**
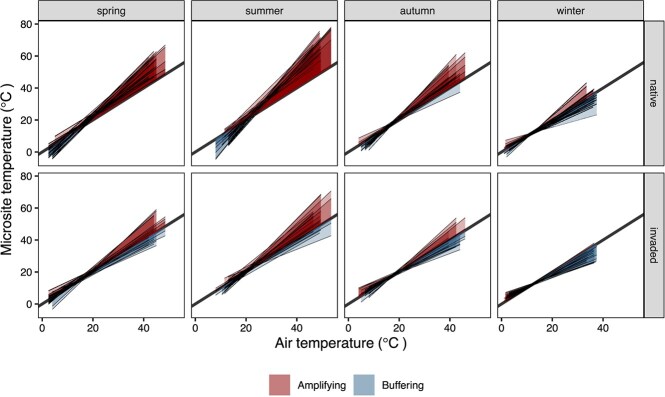
**Buffering and amplifying capacity of microsites in invaded and native areas relative to air temperature.** The plots show the linear mixed-effect models built for each combination of site, season, habitat and quadrat, with hourly ground microsite temperature as response variable, hourly air temperature as explanatory variable and day as random effect. All sites are represented, with a fitted line per quadrat (thin solid lines). The thick solid black line is the unity line. The area between a fitted line and the unity line is shaded red to indicate an amplifying effect: and blue to indicate a buffering effect (when the fitted line is above or below the unity line, respectively). A net amplifying capacity (positive values of total modifying capacity) corresponds to a situation where the red area is larger than the blue area, and a net buffering capacity (negative values of total modifying capacity) where the opposite occurs. See Fig. S9 for plots per site.

Among the four sites, daily median air temperatures ranged from 14.5 (4.5)^o^C in winter to 33.5 (4.4)^o^C in summer, with daily maximum values reaching 39.8 (5.9)^o^C (Table S23). Both native and invasive vegetation amplified air temperatures at the ground level (positive values of total modifying capacity) for most of the year except for winter, but this effect was generally more pronounced in native areas (Fig. 5). The amplifying capacity was lower in invaded areas relative to native areas, with the largest difference occurring in summer (*P* < 0.001; Table S24). By contrast, underground temperatures, at depths where *H. areolatus* nesting occurs, were generally buffered relative to air temperature throughout the year (negative values of total modifying capacity) except for some native quadrats in summer (Supplementary Fig. S10). Invasive vegetation increased this buffering capacity, especially in summer (*P* < 0.001; Table S25).

## Discussion

Changes in the thermal landscapes at the scales experienced by organisms are one mechanism underlying the impacts of plant invasions on ectothermic animal species (Garcia and Clusella-Trullas, 2019) and which can act simultaneously with ongoing warming trends (Huang *et al.*, 2014; Stark *et al.*, 2023). Our study characterized year-long changes in microclimates available to the endemic tortoise *H. areolatus* in native renosterveld habitats invaded by non-native Acacia, Eucalyptus and Pine trees. We found a mixed picture of positive and negative effects of invasive trees on the habitat’s thermal suitability to *H. areolatus*, some of which counteracted ongoing warming trends.

### Non-native areas were cooler but the effects on tortoises varied with seasons

In invaded renosterveld areas, non-native trees grew taller than the native vegetation while the understory became more homogeneous and poorer in native herbs and shrubs, similarly to other descriptions of tree-invaded fynbos vegetation (van Wilgen and Richardson, 1985). Bringing more or denser shade to an otherwise shrub-dominated habitat, the trees buffered high air temperatures and made the sites cooler, supporting our hypothesis. Although there is variation in the thermal effects of plant invasions reported in the literature (Garcia and Clusella-Trullas, 2019), similar cooling effects to those measured here have been found where native and invasive plants had contrasting growth forms (e.g. Stellatelli *et al.*, 2013; Schreuder and Clusella-Trullas, 2017). Some of the invaded landscapes previously studied became thermally less optimal for snakes and lizards (Valentine *et al.*, 2007; Stellatelli *et al.*, 2013; Carter *et al.*, 2015). Although we expected the same to occur in our study sites, the impact of invasive trees varied with season and, in some cases, was positive. Indeed, our invaded areas offered optimal temperatures (26.2–34.4°C, the species’ thermal suitability breadth B80) for longer periods than native areas in summer, but not in autumn, winter and spring. Invaded areas were also less exposed to sub-optimal hot temperatures throughout the year and had better thermal quality in the daily peak of the heat in all seasons except for winter.

Body temperature influences a range of performance indicators in terrestrial chelonians, from metabolism and food intake to locomotion and reproduction (e.g. Williams, 1981; Janzen, 1994; Mendoza *et al.*, 2023). In homogeneous habitats dominated by sub-optimal hot temperatures, *H. areolatus*’ small body size and inability to retract fully inside the shell (Loveridge and Williams, 1957; Perrin and Campbell, 1981) increase the risk of overheating and desiccation. Although individuals of *H. areolatus* spend large amounts of time taking shelter under bushes and in burrows (our personal observation), their activity time can be further restricted in such environments. This scenario likely plays out in our habitats in summer, but especially in native areas where more than half of the microsites were above B80 for an average of almost 8 h per day.

Our assessment of thermal landscapes and their suitability to *H. areolatus* hinges on the assumption that we have captured a broad enough range of temperatures to describe the thermal sensitivity of the species’ performance. Although we covered six temperature treatments in our righting trials, from 10 to 35°C, this range has not allowed us to estimate an upper temperature limit of the righting response for *H. areolatus*, and we are not aware of any published estimates. In laboratory conditions, *H. areolatus* individuals in a thermal gradient attained maximum body temperatures of 34.8°C in a previous study (Perrin and Campbell, 1981) and 33.4°C ± 4.8 for our De Tuin population collected in autumn (Morran, Garcia and Clusella-Trullas, unpublished data). These maximum body temperatures are likely different in the field, where ecological constraints are at play (Giacometti et al., 2024), and below the species’ upper thermal limits. The upper boundary of the species’ thermal suitability breadth B80 used here (34.4°C, near the maximum body temperatures recorded in the laboratory) thus provided a conservative indication of the risk of overheating.

### Non-native areas could offer refuge from warming but constrain thermoregulation

The amplifying effect of vegetation was most pronounced in summer in both habitats. Assuming a space-for-time substitution, as suggested by De Frenne (2021), this amplifying effect might thus increase with anticipated warming, bringing increased exposure to temperatures above B80 and likely above the species’ upper thermal limit. Such decrease in suitability would be more pronounced in native areas, where the amplifying effect was strongest. For robust results, the assessment of the buffering and amplifying capacity of habitats requires monitoring trends for a longer period and incorporation of other important factors such as hydrography, topography, rock structure and soil texture (Davis *et al.*, 2019; Stark *et al.*, 2023). Nevertheless, our results are in line with previous research showing stronger amplifying effect of open areas relative to closed vegetation (e.g. Chen *et al.*, 1999).

Exposure to high operative temperatures in summer, further amplified in the future, highlights the importance of behavioural thermoregulation as a strategy for *H. areolatus* to control body temperature (Perrin and Campbell, 1981). In invaded areas, however, this strategy might be hampered by the lower spatial heterogeneity of microclimates. The homogenization effect of invasive trees on vegetation is demonstrated in the literature (McKinney and La Sorte, 2007; Qian and Guo, 2010). Reduced spatial range of temperature has been recorded, e.g. in invaded temperate forests (Carter *et al.*, 2015) and was hypothesized for our invaded areas. With a narrower range of temperatures at the fine spatial scale relevant to individuals’ movement, the costs of behaviour thermoregulation increase (Huey, 1991). By contrast, our native thermal landscapes were spatially more heterogeneous, likely resulting in enhanced opportunities for small-sized ectotherms to thermoregulate by shuttling among microsites and thus better withstand warming (Sears and Angilletta, 2015; Basson *et al.*, 2017; Kemppinen *et al.*, 2024).

Both native and invaded areas offered opportunities for sheltering in cooler microclimates, an important strategy in extreme hot environments for many ectothermic species, and tortoises in specific (Sears and Angilletta, 2015; Hofmeyr and Keswick, 2018; Loehr, 2018). Although our sampling of burrows was limited, daily maximum temperature in burrows was lower than in ground microsites across the year (Fig. S11). Vegetation also provides shelter from hot temperatures and its importance for tortoises to avoid overheating has been documented in desert environments (e.g. Attum *et al*. 2013; Lagarde *et al*. 2012; McMaster and Downs 2006). In the context of global warming, invasive plants in general (McInerney *et al.*, 2021), and invasive Acacia, Eucalyptus and Pinus in Africa in particular (Wells *et al.*, 2023), can provide beneficial thermal refugia to native fauna. In fact, closed canopies of native forest can function as insulators from macroclimatic warming (De Frenne *et al.*, 2019), providing refugia for species adapted to cold environments (De Lombaerde *et al.*, 2022). The invaded areas in this study, with a predominance of microsites in the shade or partly exposed to the sun, had greater capacity to buffer high air temperatures and thus to counter the ongoing warming trend as we had hypothesized. Reliance on average temperatures (as in McInerney *et al.*, 2021) would lead us to suggest that our invasive trees could act as thermal refugia. However, inspection of other thermal metrics based on the spatio-temporal distribution of microclimates revealed negative as well as positive thermal effects. In addition to reduced spatial heterogeneity relative to native areas, invaded areas had longer exposure to sub-optimal cold temperatures (<26.2°C). To cope with winter, tortoises use strategies such as hibernation and active selection of sites more exposed to solar radiation (Lagarde *et al.*, 2002; Douglas and Rall, 2006). *Homopus areolatus* tortoises do not hibernate but change their activity pattern from bimodal to unimodal in winter (personal communication from Dr Baard). Taking advantage of their low thermal inertia, *H. areolatus* individuals are likely to spend time basking in open areas in winter to gain heat (similarly to *Homopus signatus*; Loehr, 2012, 2018) but the homogeneous vegetation cover and thermal landscapes in invaded areas might hamper this mechanism.

### Context mattered: non-native plant traits and tortoise life stage

Variation in our thermal metrics results stems from differences among the invaded quadrats, which ranged from small groups of young invasive trees to larger and denser stands of taller trees. For example, exposure to temperatures below B80 was more pronounced in invaded quadrats with denser and larger stands of invasive trees than in those in earlier stages of invasion (Fig. S12). Even if not formally captured here, these differences in quadrat characteristics and associated results expose the uncertainty and context dependency that is typical of plant invasion impacts (Martin and Murray, 2011; Garcia and Clusella-Trullas, 2019; Vitule and Pelicice, 2023). Factors such as the degree of contrast between native and invasive growth forms, the age, density and stage of invasion, the distance to the edge of the non-native tree stand and the invasive species traits influence the capacity of invasive trees to buffer ground temperatures and thus alter the habitat’s thermal quality (Martin and Murray, 2011; De Frenne *et al.*, 2019; Garcia and Clusella-Trullas, 2019; Stewart *et al.*, 2021). Longer duration of invasion by trees such as Pine and Acacia has been associated with larger negative effects on native vegetation (Holmes and Cowling, 1997; Mostert *et al.*, 2017). With widespread and advanced invasions, it is thus expected that *H. areolatus* will be exposed to highly homogeneous habitats with increased cover and associated sub-optimal cold temperatures, potentially affecting their activity time. This cooling trend in invaded areas is in contrast with the warming associated with climate change. The long-term net effect of these two opposing trends cannot be determined in this study, but the signal from the multiple metrics that influence the habitat’s thermal quality for *H. areolatus* will possibly become more consistent as invasions progress.

The thermal changes imposed by invasive trees affect *H. areolatus* individuals throughout their life cycle. Compared to adults, hatchlings and juveniles are more affected given their higher susceptibility to predation, adverse environmental conditions and unavailability of food (Segura *et al*., 2020). In turn, eggs in underground nests are influenced by changes in soil temperatures for an incubation period that can last up to 320 days (Branch, 1989) between mid-winter, when the nesting season begins, and early autumn, when hatching occurs (personal communication from Dr Hofmeyr, University of the Western Cape). As other species in the Testudinidae family (Boycott and Bourquin, 2000; Hernández-Montoya *et al.*, 2017) and in the *Homopus* genus in particular (Loehr, 2010), *H. areolatus* tortoises likely display temperature-dependent sex determination, making them vulnerable to changes in incubation conditions (Carter and Janzen, 2021). Altered underground temperature can bias sex ratios, with direct consequences for population demographics, and it also affects incubation time, probability of embryo survival and hatchling body size (de Souza and Vogt, 1994; Janzen, 1994; Leslie and Spotila, 2001). Studies with turtles and crocodiles have shown that increased vegetation cover can reduce average soil temperatures below pivotal temperatures (Leslie and Spotila, 2001) and correlate with offspring sex ratios (Janzen, 1994; Kolbe and Janzen, 2002). Daily fluctuations as well as mean temperature in nests influence sex determination and embryonic survival (Bull *et al.*, 1985; de Souza and Vogt, 1994; Georges *et al.*, 1994; Shine *et al.*, 2003; Doody *et al.*, 2006). In our invaded areas, underground conditions were significantly cooler albeit more stable than in native areas. Whether these changes will affect sex ratios and embryo development or motivate females to adjust nest selection behaviour (Doody *et al.*, 2006), however, can only be ascertained with information on the thermal tolerance of embryos, which was not available.

### Assessing the long-term balance between positive and negative effects

Temperature is a major driver of microsite selection by ectotherms (e.g. Bell *et al.*, 2021) but other ecophysiological aspects also play a role (Stewart *et al.*, 2021). In invaded renosterveld habitats, reduced density of understory lowers the availability of suitable refuges for tortoises. This change decreases protection not just from heat but also from predators and fire (Wright, 1988; McMaster and Downs, 2006; Gray and Steidl, 2015; Nafus *et al.*, 2017; Hofmeyr and Keswick, 2018), two major threats to tortoise populations (Wright, 1988; van Wilgen, 2013; Hofmeyr and Keswick, 2018; Segura *et al.*, 2020; Stanford *et al.*, 2020). The risk of predation by mammals likely increases, but detectability by birds of prey decreases with the novel tree cover (Segura *et al.*, 2020). The invasive trees studied here, especially pine species, can also increase fire risk due to their high flammability and capacity to add to fuel loads with increased aboveground biomass (Kraaij *et al.*, 2024). The presence of invasive plants has also been associated with changes in foraging by native fauna (Martin and Murray, 2011). Individuals of *H. areolatus*, with an assumed generalist diet, were observed in our study sites foraging on leaves from *A. saligna* and other invasive plants (Morran, Garcia and Clusella-Trullas, unpublished data). However, the potential for this novel food source to negatively impact individuals’ growth and health, as documented for other tortoise species (Gray and Steidl, 2015; Drake *et al.*, 2016), requires further investigation.

Whether the multiple negative and positive thermal impacts discussed here result in a threat to tortoise populations depends on the species’ traits (Martin and Murray, 2011). *Homopus areolatus*’ slow life-history strategy, territoriality and small home range (personal communication from Dr Hofmeyr, University of the Western Cape; our unpublished work) reduce the opportunities for recovery over generations or by moving spatially. At the same time, changes in invaded areas might not warrant long-lived and territorial tortoises to abandon their sites (Gray and Steidl, 2015). Benefits derived from invasive plants might constrain dispersal. The cooler temperatures of renosterveld habitats invaded by non-native trees offer *H. areolatus* individuals a way to escape overheating, which can only become more important with anticipated further warming. They can thus become ecologically trapped in invaded areas (see P. S. Stewart *et al*., 2021), but potential negative effects such as reduced opportunities for thermoregulatory behaviour, increased exposure to sub-optimal cold temperatures, altered hatchling sex ratio, higher predation risk, poorer forage quality or increased fire risk might eventually outweigh the thermal refuge benefits.

Habitat modification by invasive species has been found to negatively affect reptiles across biomes, with reported lower body condition suggesting potential reduced fitness and population decline (Macdonald *et al.*, 2023). Ultimately, quantifying the costs and benefits of plant invasions on the fitness and survival of tortoises and other native animals requires studying both behaviour and fitness changes simultaneously (Ceradini and Chalfoun, 2017) and doing so across the species’ life cycle. Our study, focused on the thermal suitability of habitats to *H. areolatus*, suggested that conserving the species hinges on preserving the native habitat while, at the same time, monitoring available thermoregulation opportunities. Under warmer scenarios, habitat engineering actions might become necessary, such as the installation of microhabitat refuges and the enhancement of incubation areas (Bonebrake et al., 2017).

In the last two decades there have been calls in the invasion biology literature to weigh the benefits as well as the costs from invasive species (e.g. Sax et al., 2022; Simberloff and Vitule, 2014). Our study, focused solely on the thermal effects of invasive trees on one resident animal species, and considering only the conservation value attached to that species, shows how complex and nuanced such assessments can be. Assessing the thermal effects of plant invasions and other global threats to biodiversity requires the integration of microclimate data with physiological responses of ectothermic organisms to thermal landscape changes as we have done here. Such mechanistic approaches are increasingly viewed as crucial for informing conservation decisions in the context of climate change and other global threats (Seebacher *et al.*, 2023).

## Supplementary Material

Web_Material_coaf016

## Data Availability

The data that support the findings of this study are openly available in the Zenodo data repository at https://doi.org/10.5281/zenodo.14863522.
