# Supplementary material for: Microclimatic changes caused by plant invasions and warming: uncovering thermal costs and benefits to a tortoise
Source: Conserv Physiol. 2025 Mar 5;13(1):coaf016. doi: 10.1093/conphys/coaf016 (PMC11884760; doi:10.1093/conphys/coaf016)
Supplement: Web_Material_coaf016 [file web_material_coaf016.zip › Garcia_Clusella-Trullas_2024_SuppInfo_microclimatic_changes_in_invaded_habitats_resubmission2025.pdf]

Microclimatic changes caused by plant invasions and warming:
uncovering thermal costs and benefits to a tortoise

Raquel A. Garcia and Susana Clusella-Trullas

SUPPORTING INFORMATION

Supplementary Methods

Measurement of tortoise carapace reflectance

We measured the reflectance of the carapace of *H. areolatus* to guide the selection of the colour paint used on the physical operative temperature models of tortoises. Using a contact probe (ASD Inc, Colorado, U.S.A.) connected to a spectroflectometer (FieldSpec® 3, ASD Inc., Colorado, U.S.A.), we measured the reflectance of the carapace of five live animals and three carcasses. We placed the probe on a lateral scute, centrally positioned, and used black putty to block the very small spaces between the sensor and the shell. For each carapace or carcass, we took three measurements. We used the mean of all measurements (0.1727; Table S1) and selected a paint with the reflectance closest to it (Plascon Super Acrylic Polvin, Code 7L017C) to paint our operative temperature models.

Table S1: Reflectance of *H. areolatus* shells. The values for each shell and carcass are means over three measurements.

Type	Mean reflectance
Shell 1	0.20464032
Shell 2	0.18636913
Shell 3	0.24877685
Shell 4	0.21430761
Shell 5	0.18867194
Mean of shells	*0.20855320*
Carcass 1	0.13640059
Carcass 2	0.10550442
Carcass 3	0.09654099
Mean of carcasses	*0.11281530*
**Mean of all**	**0.17265150**

Operative Temperature Model (OTM) validation

To validate the OTMs deployed in the field, we conducted two sets of trials. First, we compared T_e measurements of our OTMs to T_b measurements of live *H. areolatus* individuals under simulated environmental conditions. We used one large individual (217g, female) and one small individual (112g, likely a juvenile) captured in Joostenbergkloof on 26 April 2017, and 150ml can OTMs. The tests were run in a climate control room in the laboratory at 20°C. We suspended a lamp 50 cm above each tortoise and each OTM to simulate the spectrum of solar radiation (50 W halogen single-ended quartz bulb JC-BA15D with and integrated reflector for stable illumination over the 350 to 2500 nm range, Illuminator, ASD Inc., Colorado, U.S.A.). The live tortoises were secured to a wooden block using Millipore tape to ensure that they remained immobile during the trial. We inserted a probe (thin gauge type T thermocouple) in the cloaca and attached the probe to a pico logger. The physical models were also fitted with a probe inside and placed on a wooden block. After a 10 min period for habituation of the tortoise, we recorded T_e and T_b data every minute for one hour and 36 minutes. The readings showed that, once the tortoises had reached equilibrium, their temperatures were similar to those of the OTMs (Figure S1).

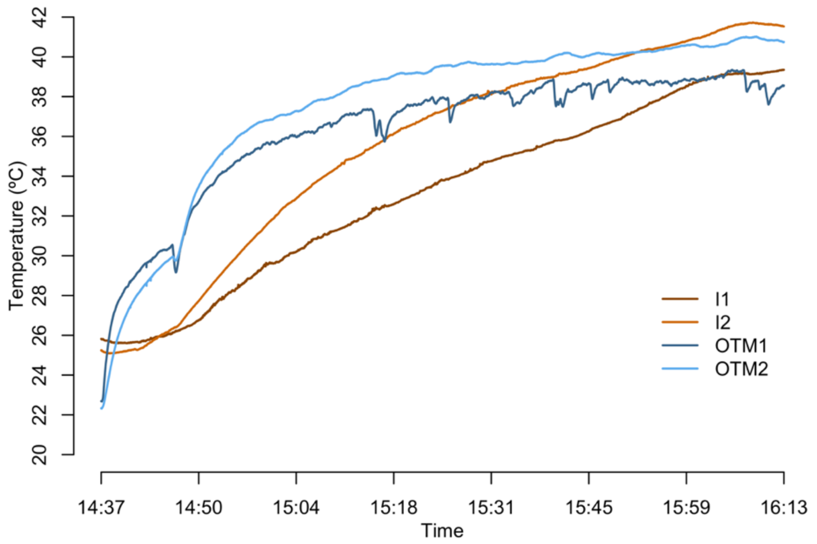

Fig. S1: Comparison of body and operative temperature. Temperature measured with thermocouples inserted in two tortoise individuals (I1 and I2) and with two operative temperature models as used in this study (OTM1 and OTM2). Once equilibrium was reached, I1's body temperature range was 39.1–39.4°C and OTM1's temperature range was 37.6–38.8°C, with a difference between the two of 0.4 to 1.6 °C; I2's body temperature range was 41.5–41.7 °C and OTM2's temperature range was 40.7–41.0 °C, with a difference between the two of 0.7 to 0.8 °C.

Second, we compared Te measurements of different physical models placed outdoors, in Joostenbergkloof. We had eight sets of models, each comprising of one shell of *H. areolatus* and one each of the small and large OTMs used in our field Te sampling. We fitted them all with iButtons inside, placed them on the ground and secured them with rope to plants or rocks, and logged temperature every five minutes for five days between 18 and 25 May 2017 (Fig. S2). We also placed one iButton inside an open white container hanging from bushes to measure air temperature. Across hours, the difference between the mean temperature of the shell and the mean temperature of any of the OTMs did not exceed 1.6 °C, and the difference between the mean temperature of the two OTMs did not exceed 1.1 °C. (Fig. S3).

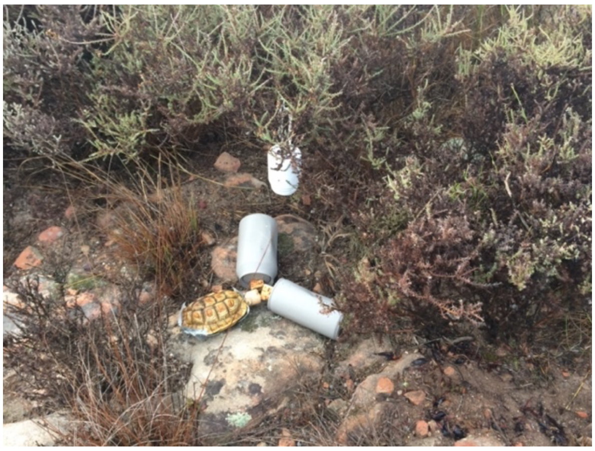

Fig. S2: Field trial set up to calibrate the operative temperature models. Eight sets of one *H. areolatus* shell and two operative temperature models, one built with a small can and the other a large can, were left in the field logging temperature every five minutes for five days.

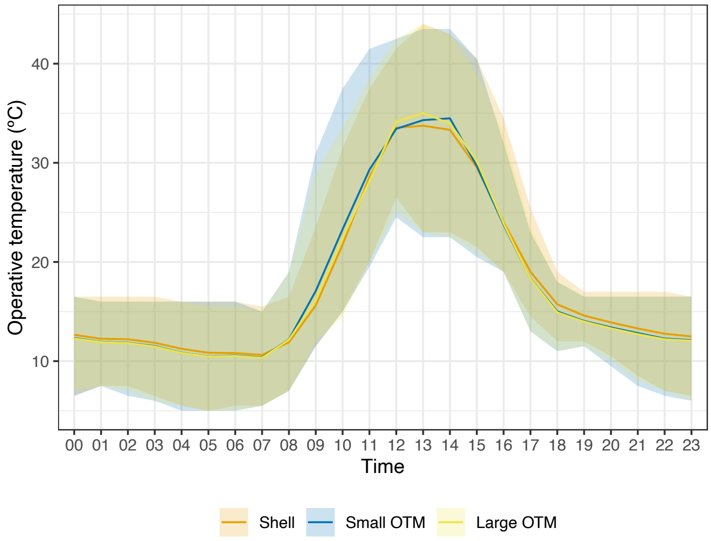

Fig. S3: Comparison of operative temperature logged by different models in the field. For each model, the hourly mean (lines) is bounded by the hourly nminimum and maximum (shaded areas) across sets and days.

Supplementary Tables

Table S2: Number of individuals used in righting response trials.

	De Tuin				Malmesbury				Both sites
**Season**	**F**	**M**	**NA**	**Total**	**F**	**M**	**NA**	**Total**	**Total**
**Spring**	9	10	3	22	8	8	2	18	40
**Autumn**	3	9	0	12	8	6	0	14	26

Table S3: Linear mixed-effect model of mean righting speed. The best linear mixed-effect model had mean righting speed as dependent variable, body temperature as fixed effect and individual ID as random factor (mean righting speed ~ poly(Tb,3) + (1|ID)). Site and season had not significant effect. The table was drawn using the "sjPlot" library (Lüdecke and Lüdecke 2015).

**Predictors**	**Estimates**	***p***	**df**
(Intercept)	0.09 (0.08 – 0.10)	**<0.001**	52.85
poly(Tb, 3)1	0.47 (0.34 – 0.59)	**<0.001**	203.59
poly(Tb, 3)2	-0.04 (-0.17 – 0.08)	0.491	196.44
poly(Tb, 3)3	-0.1 (-0.23 – 0.02)	0.114	194.26
**Random Effects**			
σ^2	0		
$\tau_{00\ ID}$	0		
ICC	0.2		
N_{ID}	57		
Observations	233		
Marginal R^2 / Conditional R^2	0.170 / 0.336		

Table S4: Number of data points for each temperature treatment in the righting response trials.

	Number of data points				
**Temperature treatment**	**De Tuin Spring**	**De Tuin Autumn**	**Malmesbury Spring**	**Malmesbury Autumn**	**Total**
**10**	3	2	6	4	15
**15**	8	5	8	4	25
**20**	10	8	7	10	35
**25**	15	11	14	11	51
**30**	22	11	10	11	54
**35**	17	11	14	11	53

Table S5: Models used to fit thermal performance curve of the mean righting response using the R package *rTPC*. The models are ordered by the AICc results, and the residual sum of squares is also included. The lowest AICc model was excluded because the shape of the curve was not biologically meaningful; our curve was thus fitted to Flinn's model (1991): rate = 1/(1 + 1 + b x temp + c x temp^2), where a is *a*, *b* and *c* are parameters that control the height of the curve, the slope of the initial increase of the curve, and the position and steepness off the decline of the curve, respectively.

Model name	Reference	AICc	Residual sum of squares
Lobry-Rosso-Flandros	Rosso *et al*., 1993	-586.5	0.0678
Flinn	Flinn, 1991	-575.8	0.0696
Briere2	Brière *et al*., 1999	-574.6	0.0696
O'Neil	O'Neil *et al*., 1972	-574.5	0.0696
Rezende	Rezende *et al*., 2019	-573.9	0.0697
Lactin2	Lactin *et al*., 1995	-573.8	0.0697
Pawar	Kontopoulos *et al*., 2018	-573.7	0.0697
Sharpeschoolhigh	Schoolfield *et al*., 1981	-573.6	0.0697
Ratkowski	Ratkowski *et al*., 1983	-573.6	0.0697
Thomas_2012	Thomas *et al*., 2012	-573.5	0.0697
Weibull	Angilletta, 2006	-573.4	0.0697
Spain	BASIC Microcomputer Models in Biology, 1982	-571.6	0.0700

Table S6: Sample sizes for thermal landscape analysis. Numbers of hours and daytime hours (07h to 19h) of logged data between 01 August 2017 and 31 July 2018 are means across OTMs.

		**Malmesbury**		**Klipheuwel**		**De Tuiin**		**Watervalle**	
**Temperature**	**Variable**	**Native**	**Invaded**	**Native**	**Invaded**	**Native**	**Invaded**	**Native**	**Invaded**
**Ground**	Number of OTMs	17	17	17	17	17	17	17	17
	Number of hours	7771	8001	8111	7792	8569	6896	8178	8220
	Daytime hours	4209	4332	4392	4219	4645	3738	4430	4455
**Underground**	Number of OTMs	2	2	3	3	3	3	3	3
	Number of hours	8160	7446	8470	7959	7894	8576	8573	7261
	Daytime hours	4419	4034	4586	4310	4279	4649	4645	3936
**Burrow**	Number of OTMs	3	2	0	0	0	0	0	0
	Number of hours	4451	3945	0	0	0	0	0	0
	Daytime hours	2412	2136	0	0	0	0	0	0
**Air**	Number of OTMs	1	0	1	0	1	0	1	0
	Number of hours	8368	0	8470	0	8575	0	8572	0
	Daytime hours	4533	0	4585	0	4648	0	4645	0

Table S7: Summary statistics for ground operative temperature composition and spatial heterogeneity. For each site, season and habitat type, the thermal composition metrics (daily median, minimum and maximum Te across quadrats) correspond to the median, minimum, maximum and range of Te across hours and days for each quadrat and then averaged across quadrats. The spatial thermal heterogeneity metrics (daily median and maximum spatial range in Te across quadrats) correspond to median or maximum range across hours and days for each quadrat and then averaged across quadrats. Daily peak range was calculated only for the hottest period of each day (the hour with maximum Te and one hour each before and after). The medians and standard deviations (in brackets) are shown. The underlying data are for all ground OTMs, from 07h to 19h between 01 August 2017 to 31 July 2018.

	Season	Habitat type	Daily median	Daily min	Daily max	Daily range	Daily median spatial range	Daily max spatial range	Daily peak median range	Daily peak max range
**Malmesbury**	Spring	Native	25.5 (0.9)	11.5 (0.0)	47.5 (3.5)	36.0 (3.3)	5.5 (2.9)	27.0 (4.6)	9.5 (5.0)	27.0 (5.0)
		Invaded	22.4 (2.9)	12.0 (0.5)	44.2 (9.2)	32.5 (9.7)	4.5 (2.7)	25.0 (6.1)	10.5 (5.8)	25.0 (8.6)
	Summer	Native	39.2 (2.1)	18.0 (0.7)	56.8 (3.8)	38.5 (3.6)	7.5 (2.9)	20.0 (5.4)	8.0 (8.2)	19.5 (7.2)
		Invaded	30.8 (3.2)	18.5 (0.7)	50.0 (8.4)	30.5 (9.2)	6.5 (3.4)	28.5 (4.0)	9.0 (4.9)	26.5 (5.3)
	Autumn	Native	24.2 (1.8)	12.2 (0.3)	41.5 (4.5)	29.8 (4.2)	4.5 (1.1)	20.0 (4.2)	10.5 (3.5)	19.5 (5.0)
		Invaded	24.0 (2.6)	13.0 (0.4)	39.0 (7.8)	26.8 (8.0)	3.7 (2.4)	24.0 (5.9)	5.0 (3.9)	22.5 (6.5)
	Winter	Native	15.1 (0.4)	7.5 (0.3)	29.0 (3.2)	21.5 (4.1)	3.0 (1.5)	20.5 (1.7)	8.0 (2.8)	20.0 (2.2)
		Invaded	14.8 (1.2)	8.5 (0.6)	28.5 (5.4)	19.5 (6.0)	2.0 (1.2)	20.5 (5.3)	3.8 (3.2)	20.5 (5.2)
**Klipheuwel**	Spring	Native	29.5 (3.1)	12.0 (1.9)	45.0 (3.2)	34.5 (4.2)	4.0 (0.8)	17.0 (4.2)	6.0 (0.9)	17.0 (4.2)
		Invaded	23.5 (1.3)	11.5 (0.3)	37.5 (4.2)	26.0 (4.7)	1.5 (1.1)	18.0 (6.4)	3.0 (2.7)	18.0 (5.3)
	Summer	Native	37.2 (3.3)	18.5 (0.6)	52.5 (3.5)	33.8 (3.2)	5.5 (1.1)	18.0 (4.1)	6.5 (1.7)	17.5 (4.0)
		Invaded	31.0 (2.5)	17.0 (1.0)	47.2 (4.5)	29.5 (4.8)	2.5 (1.5)	18.5 (3.2)	4.0 (2.0)	14.0 (3.8)
	Autumn	Native	24.0 (1.6)	12.0 (0.4)	43.5 (4.1)	30.8 (4.0)	3.0 (0.5)	17.5 (3.0)	6.0 (1.5)	17.5 (2.5)
		Invaded	23.2 (0.7)	12.5 (0.4)	37.5 (2.1)	25.8 (3.2)	1.5 (0.4)	16.0 (2.6)	3.0 (1.5)	15.0 (3.1)
	Winter	Native	15.5 (0.6)	7.5 (0.5)	29.5 (2.8)	22.5 (2.8)	1.5 (0.3)	18.0 (1.5)	4.0 (0.8)	16.0 (1.1)
		Invaded	15.8 (0.5)	9.0 (0.4)	26.0 (2.1)	17.5 (2.1)	1.0 (0.4)	14.5 (5.5)	1.5 (0.8)	14.5 (5.3)
**De Tuin**	Spring	Native	27.0 (1.4)	13.0 (0.2)	44.0 (6.0)	32.5 (5.6)	4.5 (1.6)	20.0 (1.6)	6.5 (3.9)	17.5 (3.6)
		Invaded	23.5 (1.0)	13.0 (0.4)	34.0 (3.4)	23.0 (3.8)	2.0 (0.9)	16.0 (4.8)	4.0 (1.9)	15.5 (5.3)
	Summer	Native	39.6 (2.4)	19.5 (0.2)	59.5 (2.9)	39.8 (3.2)	5.5 (3.1)	18.5 (5.2)	5.0 (5.5)	16.0 (6.0)
		Invaded	32.8 (2.9)	19.5 (1.1)	46.5 (7.0)	27.0 (5.5)	2.5 (1.0)	16.0 (7.0)	4.5 (2.1)	12.5 (8.2)
	Autumn	Native	24.8 (0.9)	13.5 (0.4)	42.0 (4.5)	28.5 (3.6)	4.5 (1.6)	16.5 (2.0)	6.5 (3.8)	16.5 (2.4)
		Invaded	24.0 (2.2)	14.5 (0.2)	33.8 (3.3)	21.0 (3.6)	1.8 (0.5)	18.5 (3.4)	3.2 (0.7)	12.5 (3.6)

	Season	Habitat type	Daily median	Daily min	Daily max	Daily range	Daily median spatial range	Daily max spatial range	Daily peak median range	Daily peak max range
	Winter	Native	16.0 (0.7)	8.0 (0.2)	30.0 (5.8)	22.0 (5.8)	2.0 (0.8)	17.0 (1.5)	6.0 (3.9)	17.0 (2.9)
		Invaded	16.0 (1.2)	9.1 (0.4)	23.5 (2.3)	16.0 (2.7)	1.4 (0.4)	11.0 (4.4)	2.0 (0.8)	10.0 (5.6)
**Watervalle**	Spring	Native	26.2 (1.7)	11.5 (0.4)	45.0 (1.7)	34.5 (1.7)	3.5 (1.2)	19.0 (2.5)	6.5 (1.8)	19.09 (2.4)
		Invaded	23.0 (2.0)	11.5 (0.2)	35.5 (7.0)	27.0 (7.2)	1.5 (1.8)	15.0 (3.7)	3.0 (2.8)	13.0 (4.0)
	Summer	Native	38.5 (2.0)	18.0 (0.7)	55.8 (1.3)	37.8 (1.8)	3.5 (1.8)	15.0 (4.1)	6.8 (3.2)	14.5 (4.3)
		Invaded	34.8 (4.7)	17.5 (0.3)	54.5 (6.9)	38.0 (7.1)	2.5 (1.9)	12.5 (5.3)	5.0 (3.9)	12.5 (5.5)
	Autumn	Native	24.0 (1.7)	13.5 (0.6)	43.0 (3.6)	30.5 (3.6)	2.0 (0.9)	14.5 (4.2)	4.0 (4.7)	14.5 (5.1)
		Invaded	22.0 (1.2)	13.0 (0.4)	30.0 (4.1)	16.5 (4.3)	1.0 (0.7)	12.0 (5.8)	2.0 (0.8)	12.0 (5.8)
	Winter	Native	15.0 (1.0)	9.5 (0.5)	30.5 (4.1)	19.0 (4.3)	1.5 (0.8)	16.0 (2.8)	3.5 (3.0)	16.0 (3.0)
		Invaded	14.5 (0.4)	9.0 (0.6)	20.0 (2.2)	9.0 (2.3)	1.0 (0.2)	14.5 (5.8)	1.5 (0.6)	14.0 (5.9)

Table S8: Linear mixed-effects model results for daily median ground operative temperature. The LMM with the best AICc score had daily median operative temperature as the dependent variable; habitat (treatment) and season as fixed effects; and quadrat as random effect (value ~ treatment x season + (1|quadrat)). The table was drawn using the "sjPlot" library (Lüdecke and Lüdecke 2015).

**Predictors**	**Estimates**	***p***	**df**
(Intercept)	38.3 (37.5 – 39.0)	**<0.001**	13819
treatmentinvaded	-5 (-5.4 – -4.6)	**<0.001**	13819
seasonautumn	-12.8 (-13.2 – -12.5)	**<0.001**	13819
seasonwinter	-22.4 (-22.8 – -22.0)	**<0.001**	13819
seasonspring	-11.3 (-11.6 – -10.9)	**<0.001**	13819
treatmentinvaded:seasonautumn	3.3 (2.8 – 3.9)	**<0.001**	13819
treatmentinvaded:seasonwinter	5.1 (4.5 – 5.6)	**<0.001**	13819
treatmentinvaded:seasonspring	2 (1.4 – 2.5)	**<0.001**	13819
**Random Effects**			
σ^2	32.11		
$\tau_{00\ quadrat}$	0.61		
ICC	0.02		
$N_{quadrat}$	5		
Observations	13829		
Marginal R^2 / Conditional R^2	0.608 / 0.615		

Table S9: Linear mixed-effects model results for daily range of ground operative temperature. The LMM with the best AICc score had daily range of operative temperature as the dependent variable;, habitat (treatment) and season as fixed effects; and quadrat as random effect (value ~ treatment x season + (1|quadrat)). The table was drawn using the "sjPlot" library (Lüdecke and Lüdecke 2015).

Predictors	Estimates	*p*	df
(Intercept)	8.3 (6.5 – 10.1)	**<0.001**	13779
treatmentinvaded	-2.5 (-2.8 – -2.2)	**<0.001**	13779
seasonautumn	-1.1 (-1.4 – -0.8)	**<0.001**	13779
seasonwinter	-2.1 (-2.4 – -1.8)	**<0.001**	13779
seasonspring	0.3 (0.0 – 0.6)	**0.035**	13779
treatmentinvaded:seasonautumn	-0.9 (-1.4 – -0.5)	**<0.001**	13779
treatmentinvaded:seasonwinter	-0.4 (-0.9 – 0.0)	0.08	13779
treatmentinvaded:seasonspring	-0.6 (-1.0 – -0.1)	**0.013**	13779
**Random Effects**			
σ^2	22.18		
$\tau_{00\ quadrat:site}$	3.97		
$\tau_{00\ site}$	2.62		
ICC	0.23		
$N_{quadrat}$	5		
N_{site}	4		
Observations	13790		
Marginal R^2 / Conditional R^2	0.102 / 0.308		

Table S10: Linear mixed-effects model results for daily minimum ground operative temperature. The LMM with the best AICc score had daily minimum operative temperature as the dependent variable; habitat (treatment) and season as fixed effects; and quadrat nested in site as random effects (value ~ treatment x season + (1|site/quadrat)). The table was drawn using the "sjPlot" library (Lüdecke and Lüdecke 2015).

**Predictors**	**Estimates**	***p***	**df**
(Intercept)	18.6<br>(18.1 – 19.1)	**<0.001**	13818
treatmentinvaded	-0.3<br>(-0.5 – -0.0)	**0.028**	13818
seasonautumn	-6<br>(-6.2 – -5.8)	**<0.001**	13818
seasonwinter	-10.5<br>(-10.8 – -10.3)	**<0.001**	13818
seasonspring	-6.4<br>(-6.7 – -6.2)	**<0.001**	13818
treatmentinvaded:seasonautumn	0.8<br>(0.5 – 1.2)	**<0.001**	13818
treatmentinvaded:seasonwinter	1<br>(0.7 – 1.3)	**<0.001**	13818
treatmentinvaded:seasonspring	0.3<br>(-0.0 – 0.6)	0.064	13818
**Random Effects**			
σ^2	11.83		
$\tau_{00\ quadrat:site}$	0.12		
$\tau_{00\ site}$	0.19		
ICC	0.03		
$N_{quadrat}$	5		
N_{site}	4		
Observations	13829		
Marginal R^2 / Conditional R^2	0.510 / 0.523		

Table S11: Linear mixed-effects model results for daily maximum ground operative temperature. The LMM with the best AICc score had daily maximum operative temperature as the dependent variable; habitat (treatment) and season as fixed effects; and quadrat as random effect (value ~ treatment x season + (1|quadrat)). The table was drawn using the "sjPlot" library (Lüdecke and Lüdecke 2015).

**Predictors**	**Estimates**	***p***	**df**
(Intercept)	55.1 (53.9 – 56.4)	**<0.001**	13819
treatmentinvaded	-7.7 (-8.3 – -7.1)	**<0.001**	13819
seasonautumn	-14.9 (-15.5 – -14.3)	**<0.001**	13819
seasonwinter	-27.2 (-27.9 – -26.6)	**<0.001**	13819
seasonspring	-11.9 (-12.5 – -11.3)	**<0.001**	13819
treatmentinvaded:seasonautumn	1.8 (0.9 – 2.7)	**<0.001**	13819
treatmentinvaded:seasonwinter	4.1 (3.2 – 5.0)	**<0.001**	13819
treatmentinvaded:seasonspring	0.9 (0.0 – 1.8)	**0.045**	13819
**Random Effects**			
σ^2	87.14		
$\tau_{00\ quadrat}$	1.82		
ICC	0.02		
$N_{quadrat}$	5		
Observations	13829		
Marginal R^2 / Conditional R^2	0.496 / 0.507		

Table S12: Summary statistics for underground operative temperature composition. For each site, season and habitat type, the thermal composition metrics (daily median, minimum, maximum and range of Te across quadrats) correspond to the median, minimum, maximum and range of Te across hours and days for each quadrat and then averaged across quadrats. The medians and standard deviations (in brackets) are shown. The underlying data are for all underground OTMs, from 07h to 19h between 01 August 2017 to 31 July 2018.

Site	Season	Habitat type	Daily median	Daily min	Daily max	Daily range
**Malmesbury**	Spring	Native	18.2 (0.4)	15.0 (0.7)	19.6 (0.5)	5.1 (0.2)
		Invaded	17.2 (1.1)	14.2 (0.4)	18.2 (1.8)	4.1 (1.6)
	Summer	Native	29.4 (1.9)	22.0 (0.7)	32.5 (2.8)	10.0 (3.5)
		Invaded	29.2 (6.7)	20.5 (0.0)	32.5 (9.2)	12.1 (9.4)
	Autumn	Native	20.0 (0.0)	17.0 (0.7)	21.1 (0.5)	3.9 (0.2)
		Invaded	20.0 (1.4)	16.6 (0.5)	20.9 (1.9)	4.5 (2.1)
	Winter	Native	13.7 (0.2)	11.8 (0.4)	14.3 (0.4)	3.0 (0.0)
		Invaded	14.0 (0.0)	12.5 (0.7)	14.6 (0.2)	2.0 (0.7)
**Klipheuwel**	Spring	Native	18.5 (0.5)	16.0 (0.6)	20.0 (1.0)	4.5 (1.3)
		Invaded	17.5 (0.6)	15.5 (0.0)	18.5 (0.6)	3.0 (0.3)
	Summer	Native	29.0 (2.4)	23.5 (0.8)	33.2 (5.0)	9.0 (6.0)
		Invaded	26.0 (1.7)	21.5 (0.8)	27.5 (2.3)	5.0 (1.5)
	Autumn	Native	21.0 (0.3)	17.5 (0.6)	23.8 (0.9)	5.0 (1.0)
		Invaded	20.5 (0.5)	18.5 (0.2)	21.0 (1.7)	3.0 (1.0)
	Winter	Native	13.6 (0.3)	12.0 (0.5)	15.0 (0.3)	3.0 (0.5)
		Invaded	14.0 (0.3)	12.5 (0.6)	15.0 (0.3)	2.0 (0.3)
**De Tuin**	Spring	Native	19.5 (0.3)	17.0 (0.8)	21.0 (0.6)	4.0 (1.0)
		Invaded	18.0 (1.0)	16.5 (1.2)	18.5 (4.2)	2.5 (5.1)
	Summer	Native	29.5 (0.5)	25.5 (0.8)	33.0 (2.0)	7.5 (2.5)
		Invaded	27.0 (1.3)	23.5 (0.5)	27.8 (2.9)	4.5 (2.3)
	Autumn	Native	21.0 (2.9)	19.5 (2.6)	22.5 (2.8)	3.5 (0.6)
		Invaded	21.0 (0.5)	19.0 (1.0)	22.0 (0.6)	2.0 (1.2)
	Winter	Native	14.0 (0.5)	12.5 (0.4)	15.0 (0.5)	2.5 (0.3)
		Invaded	14.5 (0.3)	13.5 (0.6)	15.0 (0.3)	1.5 (0.6)
**Watervalle**	Spring	Native	18.5 (1.0)	15.5 (0.3)	21.0 (2.0)	6.5 (1.8)
		Invaded	15.0 (1.2)	14.0 (1.0)	16.0 (1.2)	3.0 (0.3)
	Summer	Native	30.0 (1.3)	23.5 (1.5)	35.5 (2.9)	10.5 (2.5)
		Invaded	25.8 (1.3)	23.0 (1.3)	28.2 (2.0)	6.0 (0.9)
	Autumn	Native	21.0 (0.8)	17.5 (0.8)	23.5 (1.0)	7.0 (1.5)
		Invaded	19.5 (0.3)	17.0 (0.0)	20.0 (0.5)	3.0 (0.3)
	Winter	Native	13.0 (0.3)	12.0 (0.0)	14.5 (0.3)	3.0 (0.3)
		Invaded	13.5 (0.3)	12.5 (0.3)	14.0 (0.3)	1.5 (0.0)

Table S13: Linear mixed-effects model results for daily median underground operative temperature. The LMM with the best AICc score had daily median operative temperature as the dependent variable; habitat (treatment) and season as fixed effects; and quadrat as random effect (value ~ treatment x season + (1|quadrat)). The table was drawn using the "sjPlot" library (Lüdecke and Lüdecke 2015).

**Predictors**	**Estimates**	***p***	**df**
(Intercept)	29.6 (29.3 – 29.9)	**<0.001**	7411
treatmentinvaded	-3.2 (-3.5 – -2.9)	**<0.001**	7411
seasonautumn	-8.1 (-8.4 – -7.8)	**<0.001**	7411
seasonwinter	-15.9 (-16.1 – -15.6)	**<0.001**	7411
seasonspring	-9.8 (-10.0 – -9.5)	**<0.001**	7411
treatmentinvaded:seasonautumn	2.2 (1.8 – 2.6)	**<0.001**	7411
treatmentinvaded:seasonwinter	3.6 (3.2 – 4.0)	**<0.001**	7411
treatmentinvaded:seasonspring	1.3 (0.9 – 1.7)	**<0.001**	7411
**Random Effects**			
σ^2	9.79		
$\tau_{00\ quadrat}$	0.08		
ICC	0.01		
$N_{quadrat}$	5		
Observations	7421		
Marginal R^2 / Conditional R^2	0.726 / 0.728		

Table S14: Linear mixed-effects model results for daily range of underground operative temperature. The LMM with the best AICc score had the daily range of operative temperature as the dependent variable; habitat (treatment) and season as fixed effects; and quadrat as random effect (value ~ treatment x season + (1|quadrat)). The table was drawn using the "sjPlot" library (Lüdecke and Lüdecke 2015).

**Predictors**	**Estimates**	***p***	**df**
(Intercept)	9.6 (8.7 – 10.4)	**<0.001**	7411
treatmentinvaded	-3.1 (-3.4 – -2.7)	**<0.001**	7411
seasonautumn	-4.2 (-4.5 – -3.8)	**<0.001**	7411
seasonwinter	-7 (-7.3 – -6.6)	**<0.001**	7411
seasonspring	-4.3 (-4.6 – -3.9)	**<0.001**	7411
treatmentinvaded:seasonautumn	1.1 (0.6 – 1.5)	**<0.001**	7411
treatmentinvaded:seasonwinter	2.2 (1.7 – 2.6)	**<0.001**	7411
treatmentinvaded:seasonspring	1.4 (1.0 – 1.9)	**<0.001**	7411
**Random Effects**			
σ^2	13.65		
$\tau_{00\ quadrat}$	0.81		
ICC	0.06		
$N_{quadrat}$	5		
Observations	7421		
Marginal R^2 / Conditional R^2	0.277 / 0.317		

Table S15: Summary statistics for burrow operative temperature composition. For the Malmesbury site and each season and habitat type, the thermal composition metrics (daily median, minimum, maximum and range of Te across quadrats) correspond to the median, minimum, maximum and range of Te across hours and days averaged across OTMs. The medians and standard deviations (in brackets) are shown. The underlying data are for all underground OTMs, from 07h to 19h between 01 August 2017 to 31 July 2018.

Site	Season	Habitat type	Daily median	Daily min	Daily max	Daily range
**Malmesbury**	Spring	Native	26.0 (0.8)	19.5 (0.4)	31.0 (2.0)	11.5 (1.8)
		Invaded	28.1 (1.9)	20.8 (1.8)	31.0 (0.7)	10.8 (1.1)
	Summer	Native	30.5 (1.5)	23.0 (1.3)	34.2 (3.4)	11.0 (4.5)
		Invaded	33.8 (3.2)	23.2 (2.1)	38.0 (2.8)	14.2 (1.1)
	Autumn	Native	24.0 (2.4)	20.0 (2.8)	27.0 (3.2)	7.5 (0.9)
		Invaded	30.2 (2.5)	21.0 (0.7)	36.5 (2.8)	16.0 (2.1)
	Winter	Native	13.5 (-)	11.5 (-)	15.0 (-)	4.0 (-)
		Invaded	14.8 (0.3)	11.3 (1.1)	16.8 (0.3)	6.0 (0.7)

Table S16: T-test results for daily median burrow operative temperatures. Welch Two Sample t-tests performed on daily median operative temperature for native versus invaded areas in the site where burrow operative temperature was recorded.

Season	T	df	*p*	CT lower	CT upper
Spring	2.9	50.0	**0.006**	0.7	3.8
Summer	7.3	155.5	**0.000**	2.8	4.8
Autumn	10.4	65.4	**0.000**	6.8	10.0
Winter	2.9	31.5	**0.007**	0.3	1.8

Table S17: Linear mixed-effects model results for spatial range of ground operative temperatures. The LMM with the best AICc score had the daily values of spatial range of operative temperature at peak hour (14h, the time that was most frequently the hottest across sites) as the dependent variable; habitat (treatment) and season as fixed effects; and quadrat nested in site as random effects (value ~ treatment x season + (1|site/quadrat)). The table was drawn using the "sjPlot" library (Lüdecke and Lüdecke 2015).

Predictors	Estimates	*p*	df
(Intercept)	8.3 (6.5 – 10.1)	**<0.001**	13779
treatmentinvaded	-2.5 (-2.8 – -2.2)	**<0.001**	13779
seasonautumn	-1.1 (-1.4 – -0.8)	**<0.001**	13779
seasonwinter	-2.1 (-2.4 – -1.8)	**<0.001**	13779
seasonspring	0.3 (0.0 – 0.6)	**0.035**	13779
treatmentinvaded:seasonautumn	-0.9 (-1.4 – -0.5)	**<0.001**	13779
treatmentinvaded:seasonwinter	-0.4 (-0.9 – 0.0)	0.08	13779
treatmentinvaded:seasonspring	-0.6 (-1.0 – -0.1)	**0.013**	13779
**Random Effects**			
σ^2	22.18		
$\tau_{00\ quadrat:site}$	3.97		
$\tau_{00\ site}$	2.62		
ICC	0.23		
$N_{quadrat}$	5		
N_{site}	4		
Observations	13790		
Marginal R^2 / Conditional R^2	0.102 / 0.308		

Table S18: Linear mixed-effects model results for the thermal habitat quality index *de*. The LMM with the best AICc score had the daily values of thermal habitat quality index *de* (Hertz et al. 1993) at peak hour (14h, the most frequent hottest hour across sites) as the dependent variable; habitat (treatment) and season as fixed effects; and quadrat as random effect (value ~ treatment x season + (1|quadrat)). The table was drawn using the “sjPlot” library (Lüdecke and Lüdecke 2015).

**Predictors**	**Estimates**	***p***	**df**
(Intercept)	14.2<br>(13.4 – 15.0)	**<0.001**	13784
treatmentinvaded	-7.5<br>(-8.6 – -6.4)	**<0.001**	13784
seasonautumn	-8.3<br>(-8.6 – -8.0)	**<0.001**	13784
seasonwinter	-8.7<br>(-9.1 – -8.4)	**<0.001**	13784
seasonspring	-7.7<br>(-8.0 – -7.4)	**<0.001**	13784
treatmentinvaded:seasonautumn	5.1<br>(4.7 – 5.6)	**<0.001**	13784
treatmentinvaded:seasonwinter	8.4<br>(7.9 – 8.8)	**<0.001**	13784
treatmentinvaded:seasonspring	4.8<br>(4.4 – 5.3)	**<0.001**	13784
**Random Effects**			
σ^2	24.69		
$\tau_{00\ quadrat}$	2.88		
ICC	0.1		
$N_{quadrat}$	40		
Observations	13794		
Marginal R^2 / Conditional R^2	0.264 / 0.341		

Table S19: Time within *H. areolatus*' B80 range in native and invaded areas. Decimal hours when at least one microsite within a quadrat is within *H. areolatus*' B80 range (Hours within B80) and when more than 50% of the microsites within a quadrat are above the upper end of B80 (Hours above B80). The results presented correspond to mean [minimum-maximum] values per quadrat and day and are based on the thermal performance curve for righting response derived from the thermal sensitivity trials for *H. areolatus*.

Site	Season	Treatment	Hours within B80	Hours below B80	Hours above B80
Malmesbury	spring	native	4.8 [0.0-11.0]	7.3 [1.0-13.0]	3.7 [0.0-10.0]
		invaded	4.8 [0.0-12.0]	9.0 [0.0-13.0]	2.4 [0.0-10.0]
	summer	native	4.9 [1.0-11.0]	2.8 [0.0-9.0]	7.7 [0.0-12.0]
		invaded	7.1 [0.0-13.0]	4.2 [0.0-13.0]	5.3 [0.0-13.0]
	autumn	native	4.7 [0.0-11.0]	8.1 [2.0-13.0]	2.9 [0.0-10.0]
		invaded	4.7 [0.0-11.0]	8.7 [2.0-13.0]	2.1 [0.0-10.0]
	winter	native	1.9 [0.0-7.0]	12.5 [8.0-13.0]	0.3 [0.0-6.0]
		invaded	1.5 [0.0-9.0]	12.6 [6.0-13.0]	0.2 [0.0-5.0]
Klipheuwel	spring	native	4.8 [0.0-9.0]	7.0 [1.0-13.0]	3.6 [0.0-10.0]
		invaded	4.4 [0.0-11.0]	8.6 [2.0-13.0]	1.6 [0.0-11.0]
	summer	native	4.6 [2.0-10.0]	2.8 [0.0-12.0]	7.5 [0.0-12.0]
		invaded	5.9 [0.0-11.0]	4.1 [0.0-13.0]	5.1 [0.0-12.0]
	autumn	native	4.0 [0.0-9.0]	7.8 [1.0-13.0]	3.1 [0.0-10.0]
		invaded	3.9 [0.0-11.0]	9.0 [1.0-13.0]	1.6 [0.0-7.0]
	winter	native	2.0 [0.0-7.0]	11.8 [7.0-13.0]	0.3 [0.0-10.0]
		invaded	1.3 [0.0-6.0]	12.2 [7.0-13.0]	0.1 [0.0-8.0]
DeTuin	spring	native	5.0 [0.0-10.0]	7.1 [1.0-13.0]	3.3 [0.0-10.0]
		invaded	4.5 [0.0-11.0]	8.8 [2.0-13.0]	1.3 [0.0-9.0]
	summer	native	4.4 [1.0-10.0]	2.3 [0.0-13.0]	8.4 [0.0-11.0]
		invaded	6.0 [0.0-12.0]	3.4 [0.0-13.0]	5.5 [0.0-11.0]
	autumn	native	4.6 [0.0-11.0]	8.0 [1.0-13.0]	2.8 [0.0-9.0]
		invaded	4.4 [0.0-11.0]	8.5 [2.0-13.0]	1.4 [0.0-9.0]
	winter	native	1.8 [0.0-8.0]	12.4 [6.0-13.0]	0.1 [0.0-6.0]
		invaded	1.2 [0.0-8.0]	12.4 [6.0-13.0]	0.1 [0.0-6.0]
Watervalle	spring	native	4.2 [0.0-10.0]	7.5 [1.0-13.0]	3.4 [0.0-10.0]
		invaded	3.5 [0.0-9.0]	8.8 [2.0-13.0]	2.2 [0.0-9.0]
	summer	native	3.7 [0.0-10.0]	2.9 [0.0-13.0]	7.6 [0.0-12.0]
		invaded	4.3 [0.0-10.0]	3.5 [0.0-13.0]	6.5 [0.0-12.0]
	autumn	native	3.6 [0.0-10.0]	8.2 [2.0-13.0]	2.8 [0.0-9.0]
		invaded	3.2 [0.0-10.0]	9.5 [3.0-13.0]	1.4 [0.0-9.0]
	winter	native	2.0 [0.0-7.0]	12.0 [6.0-13.0]	0.3 [0.0-8.0]
		invaded	0.6 [0.0-6.0]	12.8 [7.0-13.0]	0.1 [0.0-7.0]

Table S20: Linear mixed-effects model results for the number of hours within the B80 range. The LMM with the best AICc score had the daily values of the number of hours when at least one microsites per quadrat as within the B80 range) as the dependent variable; habitat (treatment) and season as fixed effects; and quadrat nested in site as random effects (value ~ treatment x season + (1|site/quadrat)). The table was drawn using the "sjPlot" library (Lüdecke and Lüdecke 2015).

Predictors	Estimates	*p*	df
(Intercept)	4.4<br>(3.9 – 4.9)	**<0.001**	13818
treatmentinvaded	1.4<br>(1.1 – 1.7)	**<0.001**	13818
seasonautumn	-0.2<br>(-0.4 – -0.0)	**0.013**	13818
seasonwinter	-2.5<br>(-2.7 – -2.3)	**<0.001**	13818
seasonspring	0.3<br>(0.1 – 0.5)	**<0.001**	13818
treatmentinvaded:seasonautumn	-1.5<br>(-1.8 – -1.3)	**<0.001**	13818
treatmentinvaded:seasonwinter	-2.2<br>(-2.4 – -1.9)	**<0.001**	13818
treatmentinvaded:seasonspring	-1.8<br>(-2.0 – -1.6)	**<0.001**	13818
**Random Effects**			
σ^2	5.92		
$\tau_{00\ quadrat:site}$	0.16		
$\tau_{00\ site}$	0.22		
ICC	0.06		
$N_{quadrat}$	40		
N_{site}	4		
Observations	13829		
Marginal R^2 / Conditional R^2	0.234 / 0.280		

Table S21: Linear mixed-effects model results for the number of hours above the B80 range. The LMM with the best AICc score had the daily values of the number of hours when more than 50% of the microsites per quadrat were above the upper end of B80) as the dependent variable; habitat (treatment) and season as fixed effects; and quadrat as random effect (value ~ treatment x season + (1|quadrat)). The table was drawn using the "sjPlot" library (Lüdecke and Lüdecke 2015).

**Predictors**	**Estimates**	***p***	**df**
(Intercept)	7.8<br>(7.4 – 8.1)	**<0.001**	13819
treatmentinvaded	-2.2<br>(-2.7 – -1.7)	**<0.001**	13819
seasonautumn	-4.9<br>(-5.0 – -4.7)	**<0.001**	13819
seasonwinter	-7.5<br>(-7.7 – -7.4)	**<0.001**	13819
seasonspring	-4.3<br>(-4.5 – -4.1)	**<0.001**	13819
treatmentinvaded:seasonautumn	0.9<br>(0.7 – 1.1)	**<0.001**	13819
treatmentinvaded:seasonwinter	2<br>(1.7 – 2.2)	**<0.001**	13819
treatmentinvaded:seasonspring	0.5<br>(0.3 – 0.8)	**<0.001**	13819
**Random Effects**			
σ^2	5.82		
$\tau_{00\ quadrat}$	0.55		
ICC	0.09		
N $_{quadrat}$	40		
Observations	13829		
Marginal R^2 / Conditional R^2	0.490 / 0.534		

Table S22: Linear mixed-effects model results for the number of hours below the B80 range. The LMM with the best AICc score had the daily values of the number of hours when more than 50% of the microsites per quadrat were below the upper end of B80) as the dependent variable; habitat (treatment) and season as fixed effects; and quadrat as random effect (value ~ treatment x season + (1|quadrat)). The table was drawn using the “sjPlot” library (Lüdecke and Lüdecke 2015).

**Predictors**	**Estimates**	***p***	**df**
(Intercept)	2.7<br>(2.4 – 3.0)	**<0.001**	13819
treatmentinvaded	1.1<br>(0.6 – 1.5)	**<0.001**	13819
seasonautumn	5.3<br>(5.2 – 5.5)	**<0.001**	13819
seasonwinter	9.5<br>(9.3 – 9.7)	**<0.001**	13819
seasonspring	4.5<br>(4.3 – 4.7)	**<0.001**	13819
treatmentinvaded:seasonautumn	-0.2<br>(-0.5 – 0.1)	0.121	13819
treatmentinvaded:seasonwinter	-0.7<br>(-1.0 – -0.4)	**<0.001**	13819
treatmentinvaded:seasonspring	0.5<br>(0.2 – 0.8)	**<0.001**	13819
**Random Effects**			
σ^2	8.13		
$\tau_{00\ quadrat}$	0.42		
ICC	0.05		
$N_{quadrat}$	40		
Observations	13829		
Marginal R^2 / Conditional R^2	0.552 / 0.575		

Table S23: Summary statistics for air temperature composition. For each site, season and habitat type, the thermal composition metrics (daily median, minimum, maximum and range of Te across quadrats) correspond to the median, minimum, maximum and range of Te across hours and days. The medians and standard deviations (in brackets) are shown. The underlying data are for a single OTM, from 07h to 19h between 01 August 2017 to 31 July 2018.

Site	Season	Daily median	Daily min	Daily max	Daily range
Malmesbury	Winter	17.8 (4.5)	9.0 (3.2)	22.3 (5.6)	14.0 (6.2)
	Spring	25.2 (6.2)	14.2 (5.1)	29.5 (6.8)	14.0 (5.6)
	Summer	32.5 (5.1)	20.5 (4.2)	39.8 (5.9)	17.8 (3.9)
	Autumn	24.5 (6.1)	13.0 (2.9)	30.5 (6.7)	17.0 (5.9)
Klipheuwel	Winter	17.5 (3.7)	8.5 (3.4)	24.5 (5.1)	17.5 (6.8)
	Spring	24.0 (5.4)	11.5 (3.8)	29.0 (6.3)	18.5 (6.4)
	Summer	30.0 (4.5)	17.5 (3.1)	35.0 (5.1)	18.0 (4.7)
	Autumn	23.5 (5.3)	13.0 (3.4)	31.2 (6.3)	19.5 (6.8)
De Tuin	Winter	17.5 (4.3)	9.0 (3.5)	24.0 (6.7)	16.5 (7.6)
	Spring	24.5 (5.2)	13.5 (4.9)	29.0 (6.0)	15.5 (5.9)
	Summer	33.5 (4.4)	20.5 (3.7)	39.0 (5.3)	17.5 (5.3)
	Autumn	27.5 (6.2)	13.0 (4.3)	34.0 (6.8)	19.0 (7.1)
Watervalle	Winter	14.5 (4.5)	9.5 (4.6)	21.5 (5.0)	12.0 (4.8)
	Spring	22.0 (5.7)	11.5 (4.0)	30.5 (6.8)	20.5 (6.2)
	Summer	32.2 (4.5)	16.5 (3.8)	38.0 (4.8)	22.0 (4.5)
	Autumn	23.0 (5.1)	13.0 (3.5)	30.0 (5.8)	17.5 (5.4)

Table S24: Linear mixed-effects model results for the total modifying capacity of ground microsites. The LMM with the best AICc score had the total modifying capacity (daily values) as the dependent variable; habitat (treatment) and season as fixed effects; and quadrat as random effect (value ~ treatment x season + (1|quadrat)). The table was drawn using the "sjPlot" library (Lüdecke and Lüdecke 2015).

Predictors	Estimates	*p*	df
(Intercept)	253<br>(223.6 – 282.4)	**<0.001**	13782
treatmentinvaded	-173.3<br>(-214.9 – -131.7)	**<0.001**	13782
seasonautumn	-164<br>(-166.6 – -161.4)	**<0.001**	13782
seasonwinter	-283<br>(-285.6 – -280.3)	**<0.001**	13782
seasonspring	-74.8<br>(-77.5 – -72.2)	**<0.001**	13782
treatmentinvaded:seasonautumn	83.8<br>(80.0 – 87.5)	**<0.001**	13782
treatmentinvaded:seasonwinter	146.2<br>(142.3 – 150.0)	**<0.001**	13782
treatmentinvaded:seasonspring	30.9<br>(27.1 – 34.6)	**<0.001**	13782
**Random Effects**			
σ^2	1604.44		
$7\tau_{00\ quadrat}$	4484.43		
ICC	0.74		
$N_{quadrat}$	40		
Observations	13792		
Marginal R^2 / Conditional R^2	0.614 / 0.898		

Table S25: Linear mixed-effects model results for the total modifying capacity of underground microsites. The LMM with the best AICc score had the total modifying capacity (daily values) as the dependent variable; habitat (treatment) and season as fixed effects; and quadrat as random effect (value ~ treatment x season + (1|quadrat)). The table was drawn using the "sjPlot" library (Lüdecke and Lüdecke 2015).

**Predictors**	**Estimates**	***p***	**df**
(Intercept)	-18.6<br>(-46.9 – 9.7)	0.197	8105
treatmentinvaded	-142.5<br>(-182.5 – -102.5)	**<0.001**	8105
seasonautumn	-96.4<br>(-100.8 – -91.9)	**<0.001**	8105
seasonwinter	-157.4<br>(-162.0 – -152.9)	**<0.001**	8105
seasonspring	-139.6<br>(-144.0 – -135.2)	**<0.001**	8105
treatmentinvaded:seasonautumn	101.4<br>(95.1 – 107.8)	**<0.001**	8105
treatmentinvaded:seasonwinter	157<br>(150.4 – 163.6)	**<0.001**	8105
treatmentinvaded:seasonspring	53.8<br>(47.4 – 60.2)	**<0.001**	8105
**Random Effects**			
σ^2	2731.47		
$\tau_{00\ quadrat}$	2465.64		
ICC	0.47		
$N_{quadrat}$	24		
Observations	8115		
Marginal R^2 / Conditional R^2	0.415 / 0.692		

Supplementary Figures

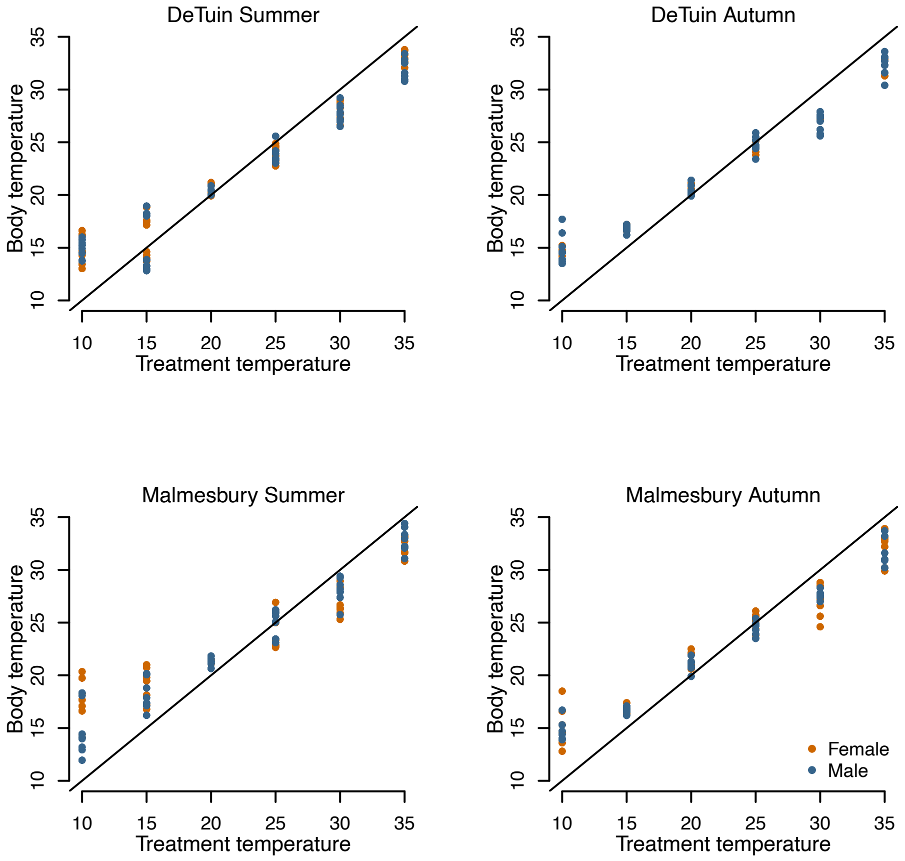

Fig. S4: Relationship between treatment and body temperature in righting response trials. Each point corresponds to an individual (blue for females, red for males) subjected to a specific treatment. Body temperature was measured in the neck fold after one hour of habituation to the treatment temperature and before the start of the trial.

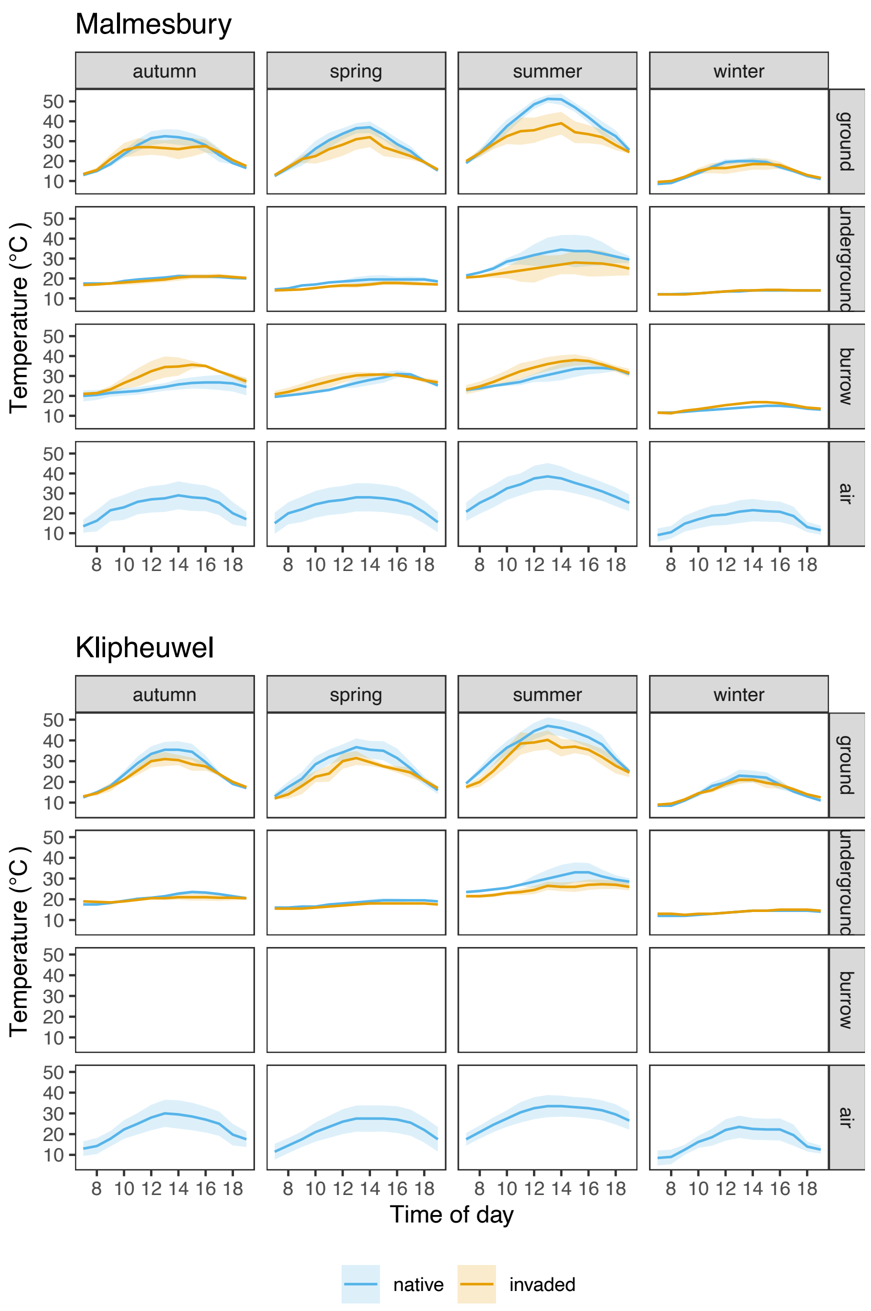

Malmesbury
Klipheuwel
autumn
spring
summer
winter
ground
underground
burrow
air
Temperature (°C)
Time of day
8 10 12 14 16 18
10 20 30 40 50
native
invaded

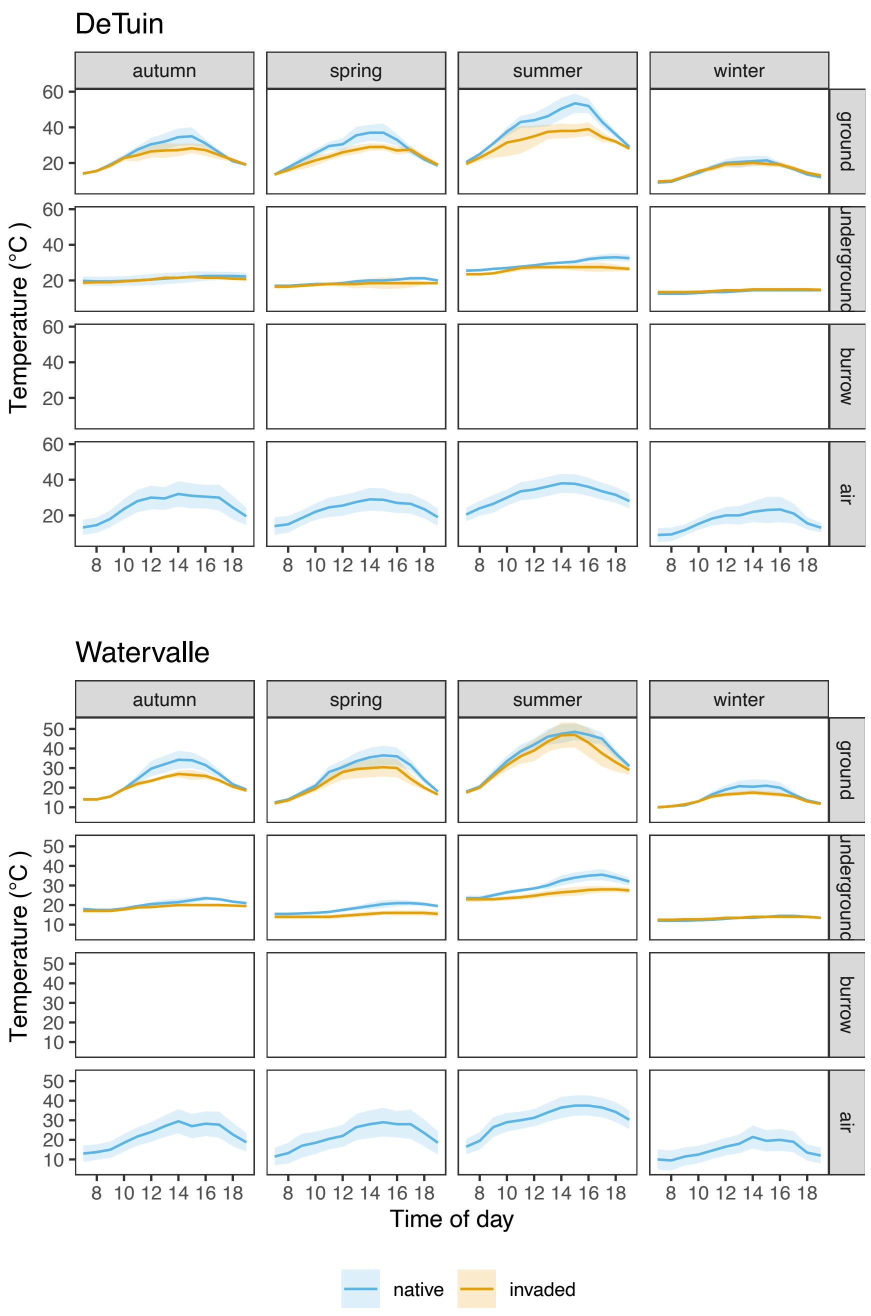

Fig. S5: Operative temperature across the day in native and invaded areas for each site. For each site, and for all seasons (columns) and for air, ground, burrow and underground temperature (rows), the plots show the median (solid blue and orange lines for native and invaded areas, respectively) and standard deviation (polygons around the lines) of hourly Te in ground and underground microsites, as well as in burrows when available. Operative temperature corresponds to hourly medians and standard deviation across all sites, quadrats and days for ground and underground microsites, across all sites, OTMs and days for burrows, and across all sites and days in the native areas for air temperature.

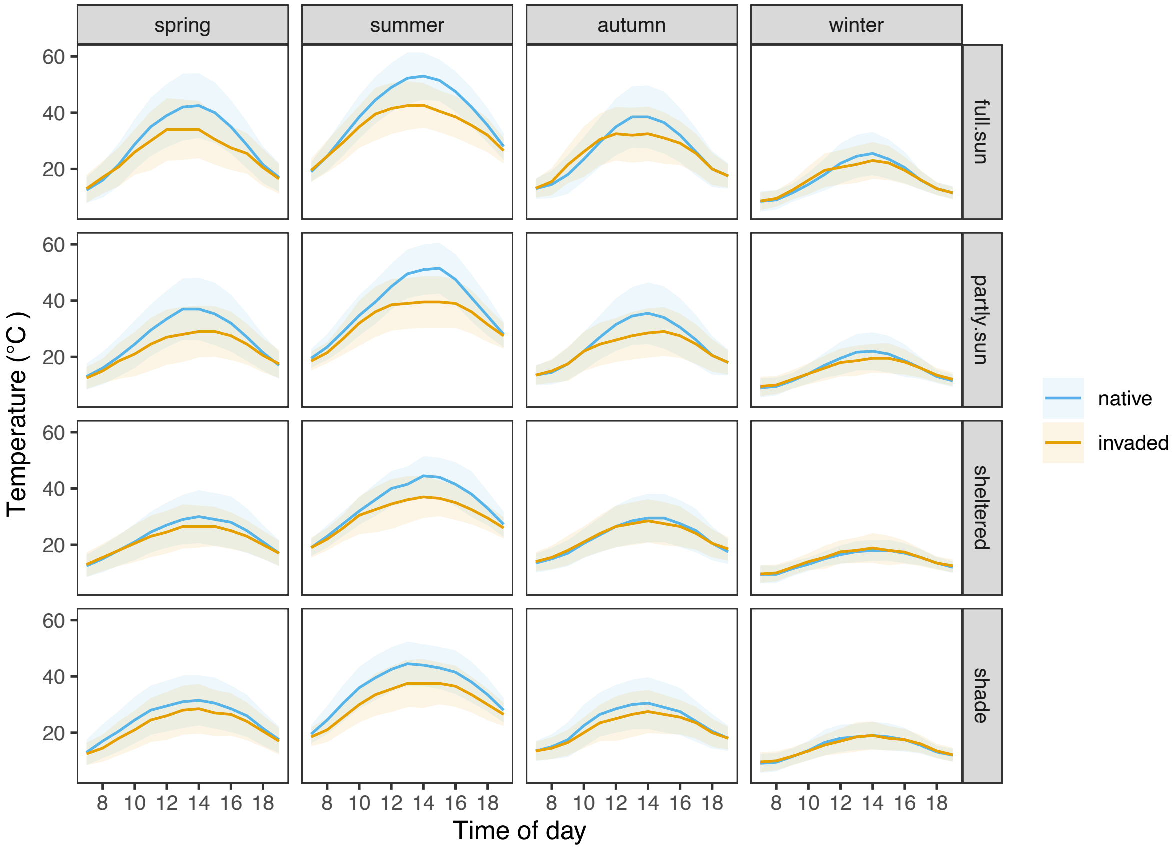

Figure S6: : Ground operative temperature in different microsite types across the day in native and invaded areas. For each season (columns), the plots show the median (solid blue and orange lines for native and invaded areas, respectively) and standard deviation (polygons around the lines) of hourly ground operative temperature in different types of microsites (rows). Operative temperature corresponds to hourly medians and standard deviation across all sites, quadrats and days.

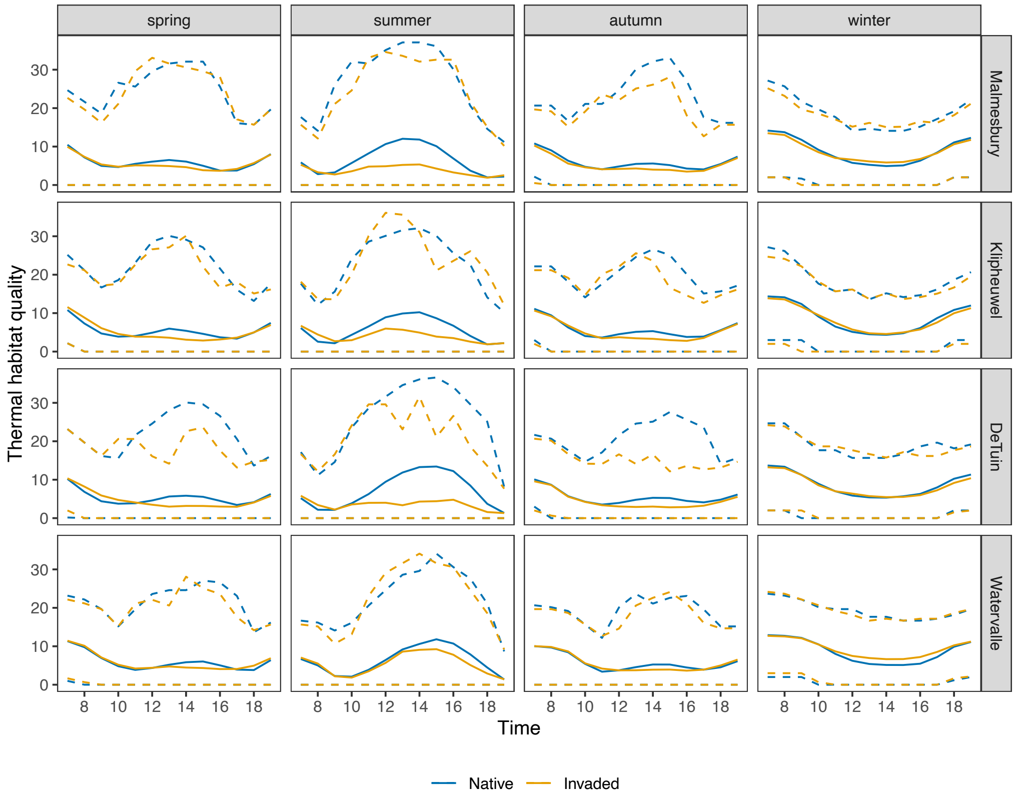

Fig. S7: Thermal habitat quality for *H. areolatus* in native and invaded areas for each site. The thermal quality index de (Hertz et al., 1993) for *H. areolatus*' is based on the B80 range derived from the thermal performance curve for the righting response of *H. areolatus*. For each site (rows) and season (columns), the mean (solid lines) and minimum and maximum (dashed lines) *de* values are shown across time for both native (blue) and invaded (orange) habitats. *de* values closer to zero correspond to higher quality thermal habitats.

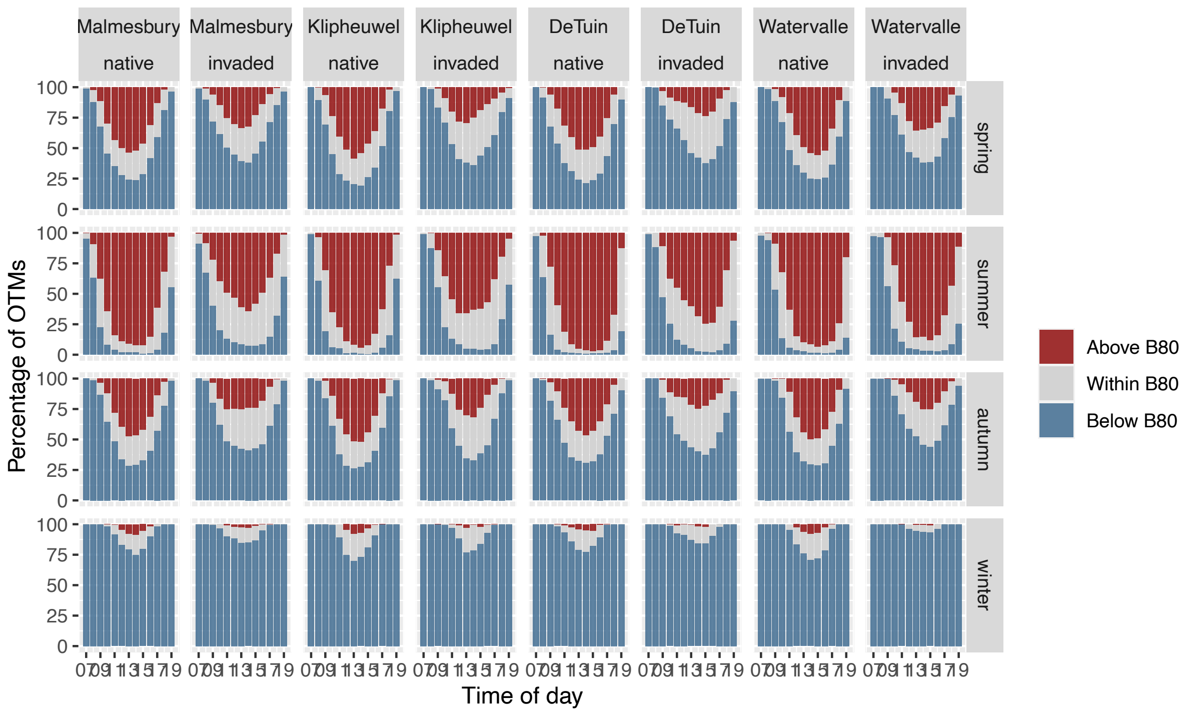

Fig. S8: Percentage of OTMs with temperature within, above and below the B80 range for *H. areolatus* per site. Percentage of OTMs with temperature within, above and below *H. areolatus*' B80 thermal suitability breadth, based on the thermal performance curve for righting response for the species, and averaged across quadrats and days.

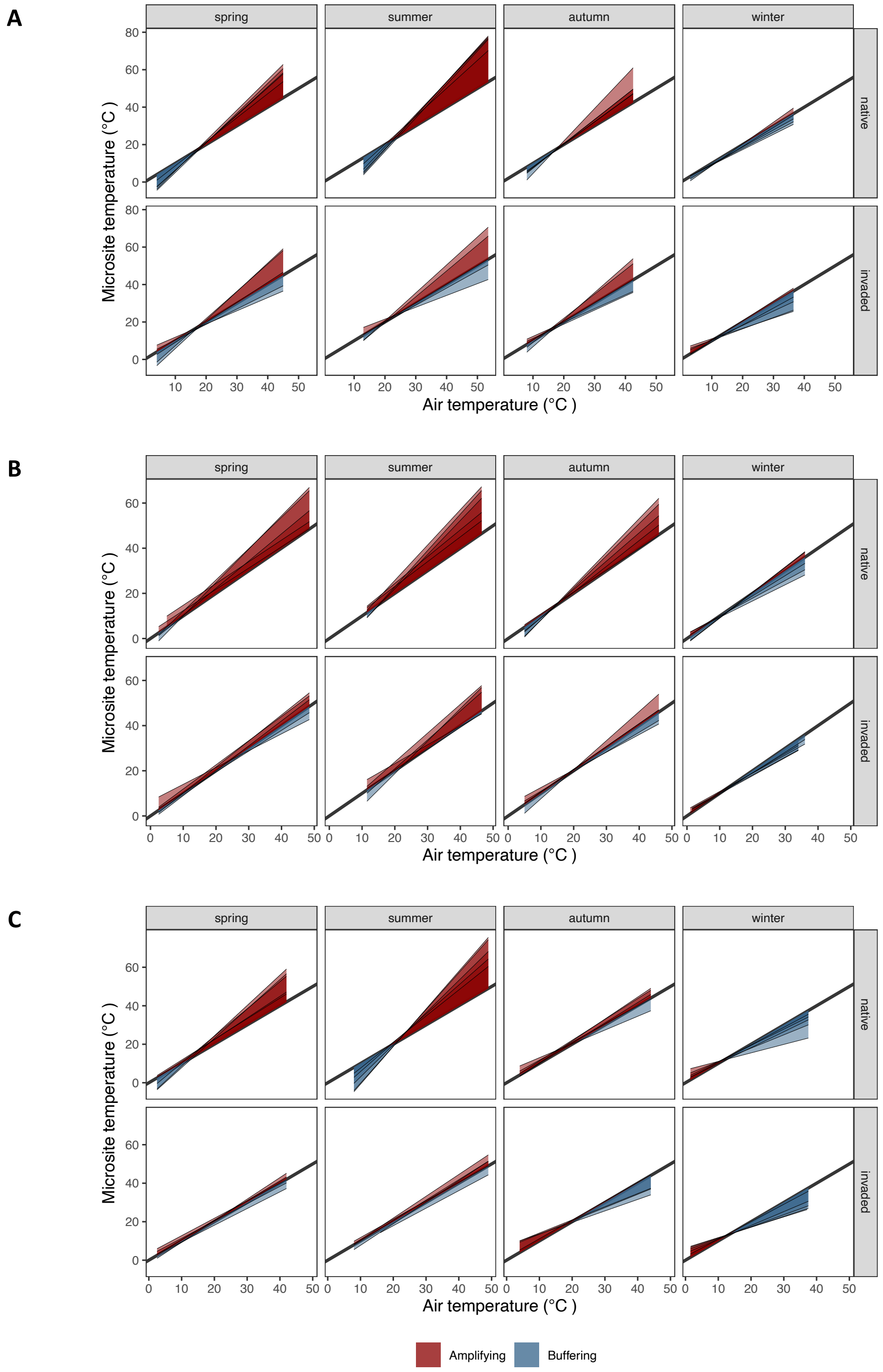
A
B
C
spring
summer
autumn
winter
native
invaded
Microsite temperature (°C)
Air temperature (°C)
Amplifying
Buffering

D

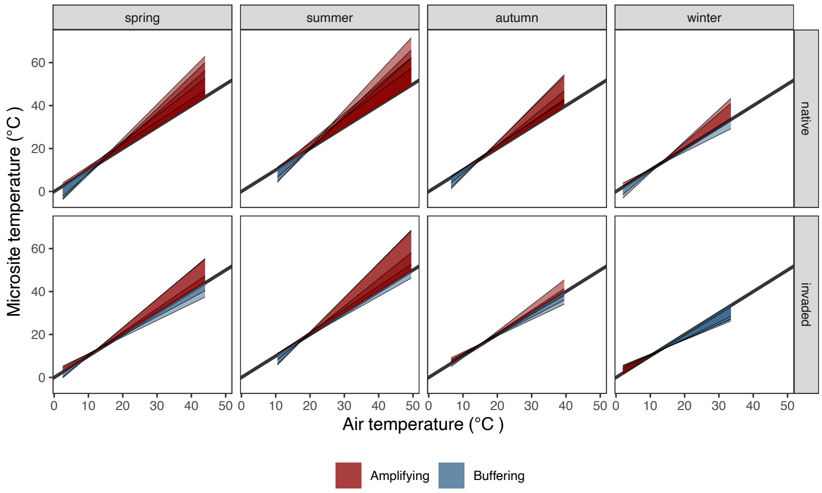

Figure S9: Buffering and amplifying capacity of microsites in invaded and native areas relative to air temperature. The plots show the linear mixed-effect models built for each combination of site, season, habitat and quadrat, with hourly ground microsite temperature as response variable, hourly air temperature as explanatory variable, and day as random effect. For each site (A) Malmesbury, B) Klipheuwel, C) De Tuin, D) Watervalle) there is a fitted line per quadrat (thin lines). The solid black line is the unity line. The area between a fitted line and the unity line is shaded red to indicate an amplifying effect and blue to indicate a buffering effect (when the fitted line is above or below the unity line, respectively). A net amplifying capacity (positive values of total modifying capacity) corresponds to a situation where the red area is larger than the blue area, and a net buffering capacity (negative values of total modifying capacity) where the opposite occurs.

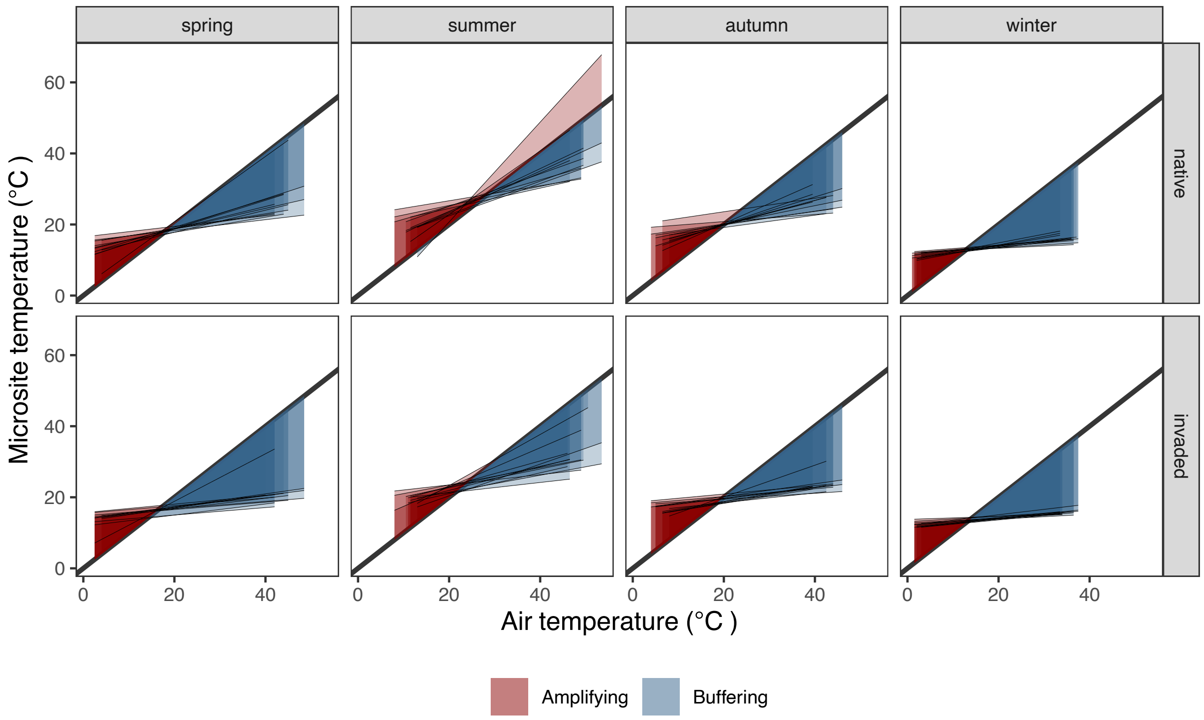

Fig. S10: Buffering and amplifying capacity of underground microsites in invaded and native areas relative to air temperature. The plots show the linear mixed-effect models that were built for each combination of site, season, habitat and quadrat, with hourly underground microsite temperature as response variable, hourly air temperature as explanatory variable, and day as random effect. All sites are represented, with a fitted line per quadrat (thin lines). The solid black line is the unity line. The area between a fitted line and the unity line is shaded orange to indicate an amplifying effect and blue to indicate a buffering effect (when the fitted line is above or below the unity line, respectively). A net amplifying capacity (positive values of total modifying capacity) corresponds to a situation where the orange area is larger than the blue area, and a net buffering capacity (negative values of total modifying capacity) where the opposite occurs.

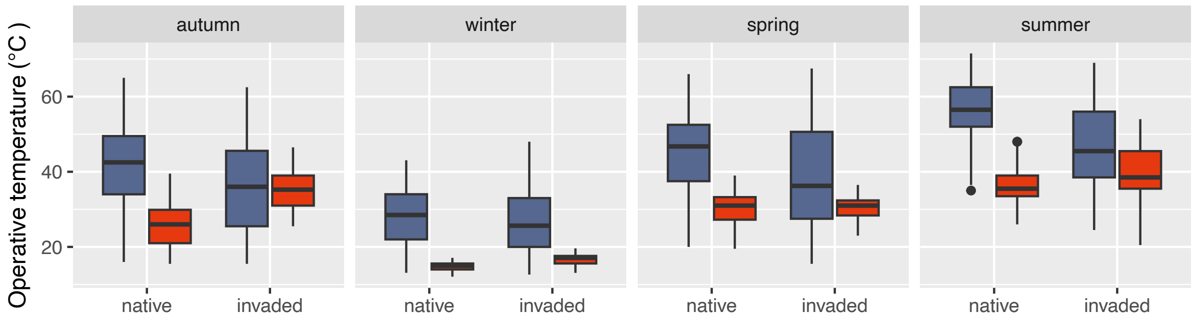

Fig. S11: Burrow and ground daily maximum operative temperatures. Daily maximum values across sites and quadrats for ground (purple) and burrow (red) microsites compared between native and invaded areas across seasons.

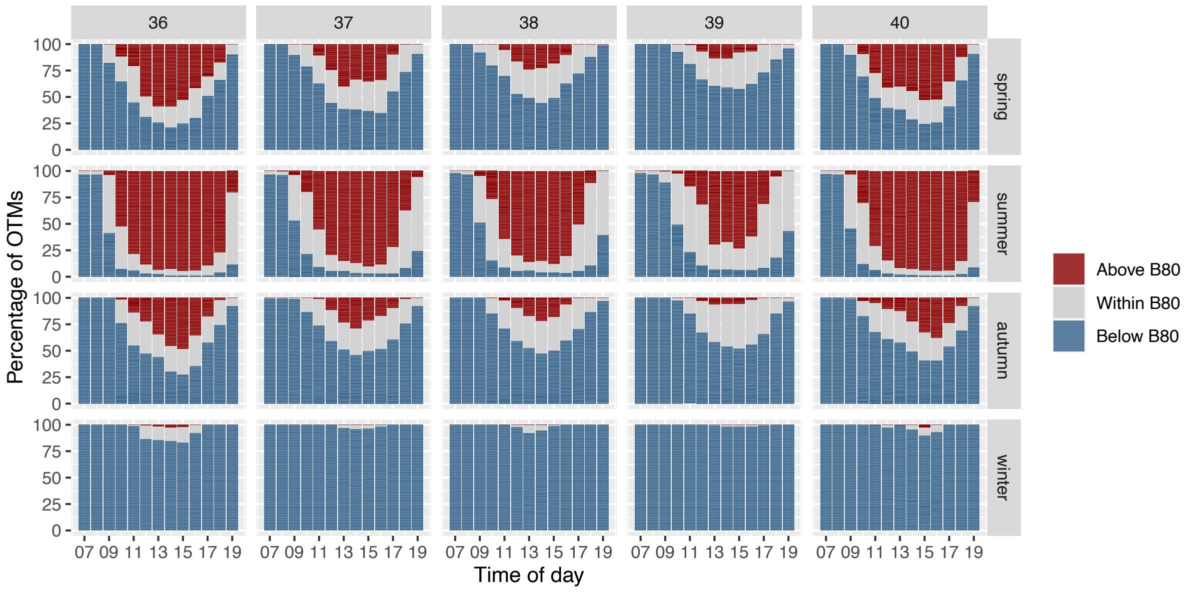

Fig. S12: Percentage of OTMs within, above and below the B80 range for *H. areolatus* for invaded quadrats in one site. Percentage of OTMs within, above and below *H. areolatus*' B80 thermal suitability breadth, for each invaded quadrat (36 to 40) in the Watervalle site and averaged across days. The values are based on the thermal performance curve for righting response for the species. Quadrat 39, the most heavily invaded (with denser stands of older Acacia trees), shows the largest percentages of OTMs below B80 and the smallest percentages of OTMs above B80. By contrast, Quadrats 36 and 40 are likely in an earlier invasion stage (with small stands of small young Acacia trees) and show the opposite trend.

References

Lüdecke D, Lüdecke MD (2015) Package 'sjPlot.' R package version 1.9.

Rosso L, Lobry JR, Flandrois JP (1993) An unexpected correlation between cardinal temperatures of microbial growth highlighted by a new model. *Journal of Theoretical Biology*, 162(4), 447-463.

Flinn PW (1991) Temperature-dependent functional response of the parasitoid Cephalonomia waterstoni (Gahan) (Hymenoptera, Bethylidae) attacking rusty grain beetle larvae (Coleoptera, Cucujidae). *Environmental Entomology*, 20, 872–876.

Brière JF, Pracros P, Le Roux AY, Pierre JS (1999) A novel rate model of temperature-dependent development for arthropods. *Environmental Entomololgy*, 28, 22–29.

O'Neill RV, Goldstein RA, Shugart HH, Mankin JB (1972) Terrestrial Ecosystem Energy Model. Eastern Deciduous Forest Biome Memo Report Oak Ridge. The Environmental Sciences Division of the Oak Ridge National Laboratory.

Rezende, E L, Bozinovic F (2019) Thermal performance across levels of biological organization. *Philosophical Transactions of the Royal Society B* 374.1778 (2019): 20180549.

Lactin DJ, Holliday NJ, Johnson DL, Craigen R (1995) Improved rate models of temperature-dependent development by arthropods. *Environmental Entomology* 24, 69-75.

Kontopoulos D-G, García-Carreras B, Sal S, Smith TP, Pawar S (2018) Use and Misuse of Temperature Normalisation in Meta-Analyses of Thermal Responses of Biological Traits. *PeerJ*. 6.

Schoolfield RM, Sharpe PJ, Magnuson CE (1981) Non-linear regression of biological temperature-dependent rate models based on absolute reaction-rate theory. *J. Theor. Biol.* 88, 719-731.

Ratkowsky DA, Lowry RK, McMeekin TA, Stokes AN, Chandler RE (1983) Model for bacterial growth rate throughout the entire biokinetic temperature range. *J. Bacteriol.* 154: 1222–1226.

Thomas, MK, *et al*. (2012) A global pattern of thermal adaptation in marine phytoplankton. *Science* 338.6110, 1085-1088.

Angilletta Jr, MJ (2006) Estimating and comparing thermal performance curves. *Journal of Thermal Biology* 31.7: 541-545.

BASIC Microcomputer Models in Biology (1982) Addison-Wesley, Reading, MA.
